# Assessing the importance of cultural diffusion in the Bantu spread into southeastern Africa

**DOI:** 10.1371/journal.pone.0215573

**Published:** 2019-05-08

**Authors:** Neus Isern, Joaquim Fort

**Affiliations:** 1 Complex Systems Laboratory, University of Girona, Girona, Catalonia, Spain; 2 Catalan Institution for Research and Advanced Studies (ICREA), Barcelona, Catalonia, Spain; Newcastle University, UNITED KINGDOM

## Abstract

The subsistence of Neolithic populations is based on agriculture, whereas that of previous populations was based on hunting and gathering. Neolithic spreads due to dispersal of populations are called demic, and those due to the incorporation of hunter-gatherers are called cultural. It is well-known that, after agriculture appeared in West Africa, it spread across most of subequatorial Africa. It has been proposed that this spread took place alongside with that of Bantu languages. In eastern and southeastern Africa, it is also linked to the Early Iron Age. From the beginning of the last millennium BC, cereal agriculture spread rapidly from the Great Lakes area eastwards to the East African coast, and southwards to northeastern South Africa. Here we show that the southwards spread took place substantially more rapidly (1.50–2.27 km/y) than the eastwards spread (0.59–1.27 km/y). Such a faster southwards spread could be the result of a stronger cultural effect. To assess this possibility, we compare these observed ranges to those obtained from a demic-cultural wave-of-advance model. We find that both spreads were driven by demic diffusion, in agreement with most archaeological, linguistic and genetic results. Nonetheless, the southwards spread seems to have indeed a stronger cultural component, which could lead support to the hypothesis that, at the southern areas, the interaction with pastoralist people may have played a significant role.

## Introduction

**Fig 1:**
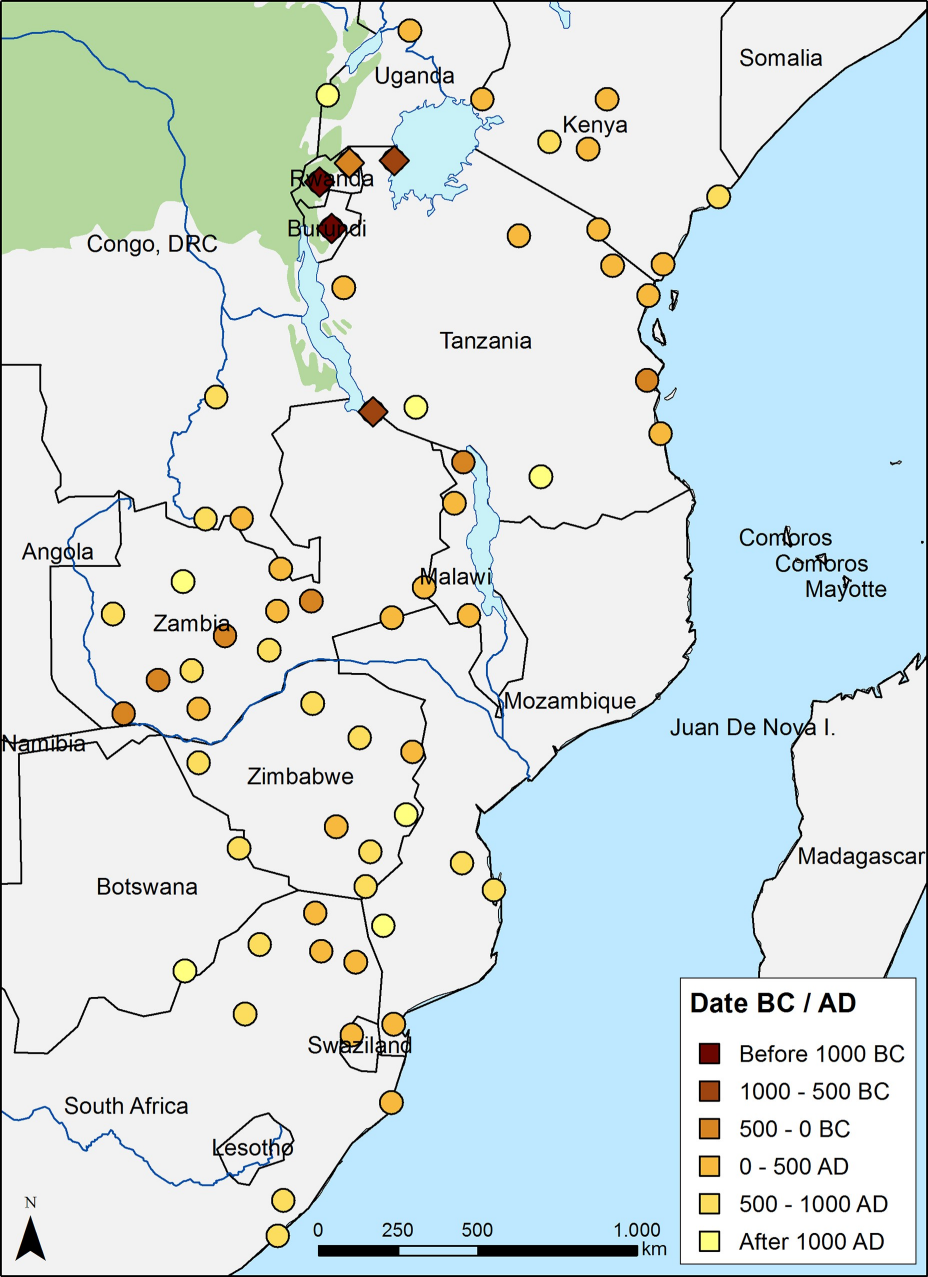
Early Bantu sites in Eastern and Southeastern Africa. The three great lakes are, from North to South, Victoria, Tanganika and Nyasa (the latter is also called Malawi). Symbols are colored according to their calibrated dates. Diamonds indicate the five sites used as possible origins of the spread. The five diamonds are, from Northeast to West and then South, Katuruka in Tanzania, Mucucu II in Rwanda, Kabacusi in Rwanda, Mubuga V in Burundi, and Kalambo Falls at the southern edge of lake Tanganika. The green color (upper left) denotes rainforest areas.

At different times and regions over the world, human populations undertook agriculture as their new way of life, gradually replacing the previous hunting-gathering economies. These processes took place with different staple crops and livestock species, but in all cases the adoption of agriculture brought radical social transformations. Often it also led to the spread of farming to neighboring regions. This was the case for the agricultural practices that appeared in West Africa and spread across most of subequatorial Africa (excluding the rainforest and southwestern Africa). In contrast with Europe, in Eastern and Southeastern Africa agriculture brought with it the first metallurgy (i.e., the Early Iron Age, EIA) [1–4]. Many authors have related the spread of farming in eastern and southeastern Africa to that of Bantu languages [1–7]. Farming and Bantu languages would have reached the western part of East Africa, i.e., the Great Lakes area (Fig 1) by the last millennium BC, from where they would have spread eastwards and southwards, reaching the southernmost areas of their spread by 400 AD [1, 3, 8].

The homeland of the Bantu languages has been placed at the area comprising the Benue Valley in Nigeria and central Cameroon [1, 2, 9, 10]. This is more than 2,000 km away from the area in which there are numerous and reliable enough archaeological dates so that spread rates can be estimated quantitatively with sufficient confidence to compare to mathematical models (see below). The proto-Bantu speaking people were stone-tool using farmers who cultivated oil palm and yams, and who may have begun to spread southward and eastward, into or around the equatorial rainforest, some 5,000 years ago [3, 10, 11]. There is currently no consensus on the paths followed by this early spread [4, 12]. Some authors advocate that the early Bantu population split into two branches, one of which would have crossed the rainforest southwards and then spread into southwestern Africa (the Western Bantu people) and the other would have spread eastwards, along the northern fringe of the rainforest, to the Great Lakes area, from where they would have spread eastwards and southwards (Eastern Bantu people) [1, 10, 13, 14]. Other authors assume that the Early Bantu people first crossed the rainforest southwards, and that then part of the population spread eastwards to the intra-lacustrine region, either along the southern fringe of the forest [5, 15] or across the rainforest itself [3, 16, 17]. These later models usually assume a subsequent split into Eastern and Western Bantu, although some authors also point toward a more complex process than a single split [12, 18].

In spite of the clear disagreement on the paths of the *early* Bantu spread, most authors do agree that the Bantu population eventually reached the Great Lakes area, from where they *later* spread into East and Southeast Africa. Also, while the first Bantu population spreading away from their homeland were stone tool using root and tree crop cultivators [1–4, 10], the Eastern Bantu were EIA people who also cultivated cereals [1–3, 9]. The Bantu people who reached western East Africa learned metallurgical, stock raising and cereal cultivation techniques, possibly from neighboring populations already established near the area and who practiced farming (Central Sudanic and East Sahelian peoples) or pastoralism (Southern Cushites) [1–3, 9]. Thus, the Bantu people developed new technological knowledge, including new farming practices, that allowed them to grow in number and spread eastwards and southwards [1–3]. Here we shall focus our analysis only on this *later* Bantu spread into eastern and southeastern Africa, for two reasons: (i) we are interested in the implications of the observed rates of spread, so we need trustable estimations of spread rates, which are possible only in areas where there is agreement on the paths of spread; (ii) the divergence in technological practices and environment clearly sets this *later* spread into the eastern subequatorial African regions apart from previous expansion processes into or close to the rainforest. It is important to note, though, that the *later* spread that we will analyze may have taken place, in fact, as two different spreads from the same origin—one eastwards and the other one southwards—rather than a single spread process [1, 3]. Below we shall assess quantitatively how the archaeological dates fit this view.

A key aspect when studying the spread of languages or innovations is the nature of the spread itself, i.e. if the spread was due to the movement of people (demic diffusion) or only the result of the spread of culture itself (cultural diffusion). In the case of the Bantu expansion, while a few authors advocate for a cultural spread [19, 20], there is agreement among many linguists, archaeologists and geneticists that this was a mostly demic process [2, 4, 7, 10, 12, 17, 21, 22] (see Table S1 in Ref. [4] for a summary). Of course, this does not imply that the spread was purely demic. For example, as mentioned above, in the intralacustrine area there was contact with other settled populations and, at some point, possibly also incorporation of individuals into the Bantu populations [1, 3]. Moreover, while 40 years ago it was assumed that the southwards spread took place demically in an area only sparsely populated by hunter-gatherers (HGs) [1], current linguistic and genetic evidence suggests the possibility of cultural interaction with pre-Bantu populations [3, 23], specially at the southern areas, where the Bantu populations may have encountered and absorbed Khoikhoi pastoralists [3, 23]. The relative importance of demic and cultural diffusion in the Bantu expansion has not been evaluated quantitatively in previous work.

Ancient genomic data relevant to the Bantu expansion are still very scarce, as only 23 individuals have been sequenced so far [24–25], and only 4 of them have been reported as farmers [25]. Moreover, all 4 farmers are from South Africa [25]. The rest are 6 HGs from South Africa, 7 HGs from Malawi, 1 HG from Ethiopia, 1 HG from Kenya, 1 HG and 1 herder from Tanzania, and 2 individuals from the coastal region of Tanzania that could not be classified [24, 25]. In the absence of ancient Bantu DNA from Western, Central and Eastern Africa, Skoglund et al. [24] assumed that a modern Mende population from Sierra Leone can be approximated to ancient Bantu farmers from West Africa and, under this assumption, showed that present-day Malawians would derive all of their ancestry from the Bantu expansion of ultimate western origin. Thus they suggested complete population replacement in Malawi but not necessarily in other regions, as their modern populations do not have only (presumed) Bantu ancestry (Fig 2D in Ref. [24]). Note that these conclusions were obtained without using any data on ancient farmers [24] and under the assumption that the modern Mende are genetically similar to the ancient Bantu. Therefore, it is not possible to assure that the same conclusions will hold when the DNA of ancient Bantu individuals from the same regions is sequenced in the future. This also implies that the percentages of Bantu and non-Bantu genomic ancestry in early farmers during the Bantu expansion has not been directly quantified yet in most regions. Indeed, only for South Africa is there DNA from ancient farmers, which is similar to that of present Bantu populations from the same area [25]. Those ancient farmers (the only African ones so far analyzed genetically) do display a non-negligible level of HG admixture (Fig 1C in Ref. [25]).

It is important to recall that the percentages of Bantu and non-Bantu genomic ancestry need not be similar to the percentages of the effects of demic and cultural diffusion *on the spread rate* (which is measured using purely archaeological data, see below). The reason is that there is no mathematical theory relating the former to the latter percentages [26]. This means that, although a purely demic spread would obviously yield a 0% non-Bantu genomic ancestry and a 0% cultural effect *on the spread rate*, a mixed demic-cultural spread could have a non-Bantu genomic ancestry above 50% and a cultural effect *on the spread rate* below 50% or vice versa. Thus, the percentages of genomic ancestry are a different problem than the percentages of demic and cultural diffusion *on the spread rate*. Here we deal with the latter problem (the formal definition of the cultural effect *on the spread rate* will be given in Eq (2) below).

It is also important to stress that knowledge of the percentages of Bantu and non-Bantu ancestry in the genomes of early farmers involved in the Bantu expansion does not make it possible to quantify the number of hunter-gatherers that were incorporated into the farming communities per pioneering farmer and generation (i.e., the intensity of cultural diffusion *C* [26]) because, again, there is no mathematical theory relating both quantities. Indeed, it has been shown that the intensity of cultural diffusion *C* can be estimated from the archaeological spread rate [26] or, alternatively, from ancient genetic clines [27], but not from the percentages of genomic ancestry (i.e., the coefficients in an expansion of the statistic *f*_4_, which is a variance of gene frequencies) or other genetic measures (as used in admixture analysis, principal components, structure analysis, D-statistics, etc.) [27]. This is due to the fact that the dynamics of different genetic markers are driven by different processes in addition to cultural diffusion (drift, selection, etc.), so it is not possible to analyze many markers (e.g., genome-wide data) under the assumption that only demic and cultural diffusion were important. Such an assumption can be made only for very specific markers [27].

In contrast to the percentages of genomic ancestry (and other genetic measures used by the methods mentioned above), the archaeological spread rate is directly related to the intensity of cultural diffusion *C* (the mathematical relationship will be given in Eq (1) below). It has been shown previously that this makes it possible to estimate the predominance of demic or cultural diffusion by comparing the archaeological spread rate of farming fronts to the results from demic-cultural wave of advance models [26, 28, 29]. In Europe, such an analysis predicted that cultural diffusion was less important than demic diffusion [26], in agreement with the later widely accepted result (from ancient genome-wide data) that the spread was mostly demic [30]. In southwestern Africa, in contrast, the analysis of the rate of spread inferred from archaeological remains suggested that cultural diffusion was the driving mechanism for the spread of Khoikhoi pastoralist populations [29]. This result agrees with the main archaeological view of the process, and it also led to the hypothesis that transitions into pastoralism may have had a stronger cultural component than transitions into farming [29]. Therefore, a strong analytical method [26] is available that enables the quantification of the predominance of demic or cultural diffusion processes from archaeological data.

In this paper we will apply this methodology to assess the relative importance of demic and cultural diffusion processes in the spread of the Bantu people across East and Southeast Africa. Geostatistical analyses of the Early Bantu archaeological database compiled by Russell et al [4] will allow us to infer quantitatively the most probable region and date of origin of the Bantu spread into southeastern Africa, as well as the average rate at which this spread took place. By comparing these results to the predictions from the demic-cultural wave of advance model mentioned above [26] we shall estimate the nature of the spread process, a result that we will discuss against the current views on the Bantu spread in southeastern Africa.

## Materials and methods

### 1. Database

We will use archaeological data from the EIA database published by Russell et al [4], which they prepared from a more extensive database of sub-Saharan EIA sites [31] by selecting only the dates of the earliest occupations. Russell et al [4] reported the dates of 107 sites in their Table S2. Of these, 74 samples (69%) were charcoal, 3 (3%) were wood, 1 (1%) was honey, 1 (1%) an ash layer, 1 (1%) human bone collagen/charcoal, and 27 samples (25%) were of unknown type (meaning that T. Russell could not source what material had been dated). The table 'Sample type' in our S1 Data includes the kind of material dated for each site.

The database by Russell et al [4] comprises the whole area of the Bantu spread, but as we have explained in the introduction, we will only analyze the spread in eastern and southeastern Africa (because the routes are less controversial and the spread rates can thus be estimated with confidence). Therefore, in this paper we shall use only 70 of the original 107 dates that fall in the area corresponding to the eastern half of the subcontinent (see S1 Data), which is often linked to the eastern Bantu spread in the literature [1, 18, 32, 33]. We have audited all of the apparent outliers in the database and rejected six sites that present dates significantly earlier than the currently accepted chronologies and/or that have previously been assessed as being unreliable or corresponding to pre-Bantu populations (see Section A in S1 Text for details on the six sites that we have excluded from the analysis). Thus we have a database of 64 early Bantu/EIA sites (Fig 1) with which we shall analyze the Bantu spread in eastern and southeastern Africa (see S1 Data).

### 2. Time-space regressions

When assuming that a geographical spread process took place gradually from a relatively small origin region, we can estimate the average rate of this spread through time-space linear regressions. To do so we plot the mean calibrated dates of sites and their distances from the assumed origin of the spread; here we will consider the five sites represented as diamonds in Fig 1 as possible origins (as discussed below). We use calibrated dates Before Present (cal BP) in our plots rather than cal BC/AD dates because the former is a continuous scale for the studied period (the millennia before and after the turn of the era), i.e. it is not affected by the nonexistence of a year 0 BC/AD. Distances to each possible origin are measured as great-circle distances, i.e. as the shortest distance along the Earth surface if assumed a sphere (of radius 6371 km), which we compute from the sites geographical coordinates (latitude and longitude) applying the Haversine formula [34] (we include all computed distances in S1 Data). When fitting the data to a straight line, we obtain a correlation coefficient (*r*) that indicates the goodness of the fit, and thus, how appropriate it is to describe the process as a constant-speed spread from the assumed origin. The source yielding the highest absolute value of *r* will be the most probable source of the spread [29, 35, 36]. Since the slope of a time-space regression has units of time over space, we estimate the average rate of spread from the slope as its inverse, *s* = −1/*slope* (the minus sign is needed as a result of using BP dates, which increase backwards in time). We could in principle obtain the speed more directly from space-time regressions than from time-space regressions. However, because archaeological dates are usually known with more uncertainty than locations, the error introduced into the speed estimate is lower when applying time-space regressions [37]. As in previous work [29], the 80% CL error of the slope (*σ*_80% *slope*_) is obtained by multiplying the standard error of the slope by the value of Student's *t-*distribution for a 80% CL (*t*_.90_) and *M*-2 degrees of freedom, where *M* is the number of sites [29]. We also obtain the 80% confidence level (CL) error for the speed, which we compute from the 80% CL error of the slope applying error propagation theory as *σ*_80% *speed*_ = *σ*_80% *slope*_/(*slope*)^2^ [29]. In Section D in S1 Text we show that using a 95% CL the conclusions are the same (but in the main paper we use a 80% CL to make direct comparison to Ref. [29] possible).

### 3. Demic-cultural wave of advance model

Cultural changes, such as the adoption of agriculture, may spread as a result of population movement (demic diffusion), by cultural transmission to other populations (cultural diffusion), or more generally, as a combination of both processes with different relative importance. It has been shown that is possible to assess which process played a major role by comparing the observed rates of speed to those predicted by a demic-cultural model [26]. In this paper we compare the speeds of the Bantu spread obtained by regression (previous section) with those predicted by a wave of advance model that includes demic and cultural diffusion [26]. The predicted front speeds are given by [26]

$$s=\min_{\lambda >0}\frac{aT+ln[\left(1+C)(\sum_{i=1}^{N}p_{i}I_{0}(\lambda r_{i})\right)]}{\lambda T},$$ (1)

where *a* and *T* are the reproduction rate and the generation time of the farming population. Cultural diffusion is included by means of parameter *C*, which is equal to the number of non-farmers (per pioneering farmer and generation) that adopt agriculture. *C* is called the intensity of cultural diffusion [26]. Demic diffusion is included by means of the summation in the last parentheses, which takes into account the probabilities *p*_*i*_ of the farmer population to disperse a distance *r*_*i*_ (for *N* possible distances). These distances and probabilities are estimated using histograms obtained from ethnographic observations (more details and specific values will be given in Results and Discussion, subsection 3), and *r*_*i*_ can be defined as the distance between parents and children (e.g., between the birthplace of an individual and one of her/his children) [26]. The function $I_{0}(\lambda r_{i})=\frac{1}{2\pi }\int_{0}^{2\pi }exp[-\lambda r_{i}cos\theta ]d\theta$ is the modified Bessel function of the first kind and order zero. *λ* is a positive parameter related to the shape of the front (more precisely, if *r* is the radial distance to the origin of the population spread, and *z* = *r*−*st* is the distance measured in a frame moving with the front, the density of farmers is *N* = *N*_0_*exp*[−*λz*] for *z*→∞) [26]. In order to determine the front speed *s*, we plot the fraction in Eq (1) as a function of *λ* for given values of *a*, *T*, *C*, *p*_*i*_ and *r*_*i*_ (*i* = 1,2,…,*N*), and the speed *s* is the minimum in the plot [26].

Comparing the speeds predicted by Eq (1) to the observed range, we can estimate the range of *C* that better fits the observations in the case of the Bantu spread. In order to estimate the range of the cultural effect (percentage of cultural diffusion) implied by this range of *C* we apply the following definition [26]

$$\%cultural\;effect=\frac{s-s_{demic}}{s}\cdot 100,$$ (2)

where *s* is the speed predicted by Eq (1) for a given value of the intensity of cultural transmission, *C*, and *s*_*demic*_ is the front speed predicted by a purely demic model, i.e. by Eq (1) when *C* = 0. Similarly to Eq (2), the percentage of the demic effect is $\frac{s_{demic}}{s}∙100$. Obviously, the sum of the cultural and demic effects is always 100%.

## Results and discussion

### 1. Overall rate of spread

To quantify the spread rate from the Great Lakes area by means of regression analysis, we first need to choose an origin for this spread. The earliest dates in our database (once we have rejected clearly pre-Bantu dates; see Section A in S1 Text) fall west and south of Lake Victoria (see the darkest diamonds in Fig 1), thus we shall use these sites as possible origins of the expansion in our regression analyses. The two oldest dates—Mubuga V (1422 cal BC) in Burundi and Kabacusi (1209 cal BC) in Rwanda—correspond approximately to the time of the earliest arrival of Bantu speaking people in the area [3, 6, 9]. However, it is usually considered that the spread away from the intralacustrine region took place some centuries later [1, 3]. Therefore, we will also study as possible origins of the spread the next two oldest sites in the same area—Katuruka (585 cal BC) in northern Tanzania and Mucucu II (477 cal BC) in Rwanda (see Fig 1). Finally some authors, advocating for a ‘late split’ of the Bantu languages, set the origin for the southwards expansion west of Lake Tanganyika, at the Democratic Republic of Congo [12]. However, the database does not contain any old dates from this area (see Fig 1, S1 Data and Ref. [4]), thus the nearest option that we may consider as possible origin of a southwards spread is the site in Kalambo Falls (878 cal BC), located at the southern edge of Lake Tanganyika (southernmost diamond in Fig 1).

In several case studies, it has been shown that the arrival of agriculture led to substantial population growth [38]. In this sense, agriculture has been often described as an 'advantageous' cultural trait. However, agriculture often spread together with additional, non-advantageous (or less advantageous) traits, such as language or neutral genetic markers. Such traits are called 'hitchhiking' traits [39] (more generally, cultural hitchhiking refers to changes in a population feature, e.g. genetic diversity, under the influence of culture [40]). In the Bantu expansion, well-known hitchhiking traits include Bantu languages, ceramics and (in eastern and southeastern Africa) Iron metallurgy.

Strictly, if agriculture is the trait used to measure the spread of the Bantu populations, ideally we would like to use dated samples directly related to agriculture (especially for the five origins suggested above). Unfortunately, however, this is seldom possible, because sites in the database published by Russell et al [4] have rarely a date on a domestic crop for example. Thus, Russell et al [4] had to use proxies to assign almost all dates to Bantu populations. For example, for the site Mubuga V (the first of the five possible origins introduced above) the date was obtained from charcoal associated with a very fine pottery of Early Iron Age topology [41]. For Kabacusi (the second origin considered above) the material dated was a piece of charcoal extracted from scoria collected on the ground surface, and charcoal dated from a slag furnace yielded a somewhat later date [41]. Katuruka (the third origin above) is a site with 'dimple-based' pottery (which is characteristic of the Bantu Early Iron Age) associated with brick-built iron-smelting furnaces [42]. Similarly, for the Mucucu II/3 lioness shelter (the fourth origin above) the sample dated was charcoal associated with pottery [43]. Finally, Kalambo falls (the fifth origin above) was dated by means of scattered charcoals immediately beneath an Iron Age midden [44]. These examples show that in practice, it is seldom possible to use material directly related to agriculture to assign dates to Bantu Iron Age populations. Certainly, it would be much better to use samples directly related to agriculture, but unfortunately this is not possible with the data available to us at present. However, the assignations made by Russell et al [4] seem reasonable.

We apply regression analysis to the 64 sites in the database for each of the 5 possible origins, to assess their validity as possible sources of the spread and estimate the average speed. However, the results yield very low correlation coefficients, below |*r*| = 0.40, whichever the assumed origin (see Table 1, row 'All data'). Observing Fig 1 we can see that several sites are substantially later (lighter color) than others in surrounding areas. Note also that several sites in central Africa display dates rather later than even the earliest arrival at the furthest distances to the South. Obviously, these observations imply that, while each date in the database may indeed indicate the first presence of Bantu populations *in a given site*, it does not necessarily correspond to the earliest arrival of Bantu populations in the area *surrounding the site considered*. Because we want to estimate the speed of the propagating front at the continental scale, we should not include in our analysis dates corresponding to later local dispersals. Therefore, similarly to the approach followed in a previous analysis of the Khoikhoi herding spread in Southwest Africa [29], we shall repeat the analysis but with a database including only the oldest sites in each region (see Section B in S1 Text, especially the interpolation maps in Figure C in S1 Text). This reduces the database to 31 sites (indicated as “Early spread” in S1 Data) but yields substantially better results. Indeed we can see from Table 1 (row 'Oldest data') that now the correlation coefficients are much higher, with |*r*| = 0.65 in the best cases. We see also that the best results are obtained when considering the origin of the spread taking place at the older dates (Table 1, columns Mubuga V, Kabacusi and Kalambo Falls) rather than at the mid last millennium BC, implying that the spread within the intralacustrine area and away from it can be considered a single process.

**Table 1:** Absolute correlation coefficients obtained for five possible origins of the spread and different datasets. Values close to 0.8 or higher are shown in bold. The parentheses around the Eastern data results from Kalambo falls indicate that this site would not in principle correspond to the Eastern spread, due to its location to the South of the area West of lake Victoria (Fig 1).

	Mubuga V (1422 cal BC)	Kabacusi (1029 cal BC)	Kalambo Falls (878 cal BC) *	Mucucu II/3 (477 cal BC)**	Katuruka (585 cal BC)**
All Data	0.39	0.38	0.25	0.24	0.24
Oldest data	0.65	0.63	0.65	0.43	0.48
Southern data	0.43	0.44	0.29	0.25	0.24
**Oldest southern data**	**0.87**	**0.86**	**0.75**	**0.80**	**0.81**
Eastern data	0.54	0.50	(0.34)	0.32	0.27
**Oldest Eastern data**	**0.84**	**0.79**	**(0.79)**	0.60	0.66

* The results are computed without considering the two oldest dates (Mubuga V and Kabacusi).

** The results are computed without considering the three oldest dates (Mubuga V, Kabacusi and Kalambo Falls).

Although the correlation coefficients obtained using only the oldest dates are high, they are still too low to obtain reliable estimates of the average speed of the spread process. Nonetheless, we can obtain a first estimate of the range of speeds that we can expect. From the regression using Mubuga V as origin we obtain that the observed rate of spread is 2.46±0.70 km/y at the 80% confidence level (CL), for an origin at Kabacusi the speed is 2.71±0.81 km/y at the 80% CL, and for an origin at Kalambo Falls it is 2.20±0.66 km/y at the 80% CL (the latter result has been obtained excluding Mubuga V and Kabacusi, since they are substantially earlier dates). Therefore, this analysis indicates that the average rate of spread of the Early Bantu population was probably in the range 1.5–3.5 km/y (80% CL). Although this range has been obtained for still relatively low values of the correlation coefficient (|*r*| = 0.63–0.65, see Table 1), it is interesting as a first qualitative approach. In particular we see that this range of speeds is relatively fast, at least when compared to the range 0.9–1.0 km/y obtained for the spread of the Neolithic in Europe (also by regression of calibrated dates versus great-circle distances) [36]. However, it is substantially slower than some previous estimates (obtained with fewer data) according to which the Bantu expansion took place at rates of 9–15 km/y [2,45].

### 2. Southwards and eastwards spreads

The Bantu population spread from the Great Lakes region into East and Southeast Africa, but this did not necessarily happen as a single process. In fact, although all Bantu populations in this area belong to the eastern Bantu linguistic group, the eastwards and southwards spreads have been considered as two different processes on the basis of archaeological and linguistic data [1, 3]. For this reason, we shall try to improve the result obtained in the previous section by dividing the data into two subgroups, corresponding to the eastern and southern spreads. We consider as belonging to the eastern spread all sites east of Lake Tanganyika and Lake Nyasa (Fig 1), and to the southern spread all sites south of Lake Tanganyika and west of Lake Nyasa (see S1 Data). We include the sites in the region west of Lake Victoria (where the oldest sites are found) in both spread processes, except when assessing a possible southwards spread from Kalambo Falls (south of Lake Tanganyika).

**Fig 2:**
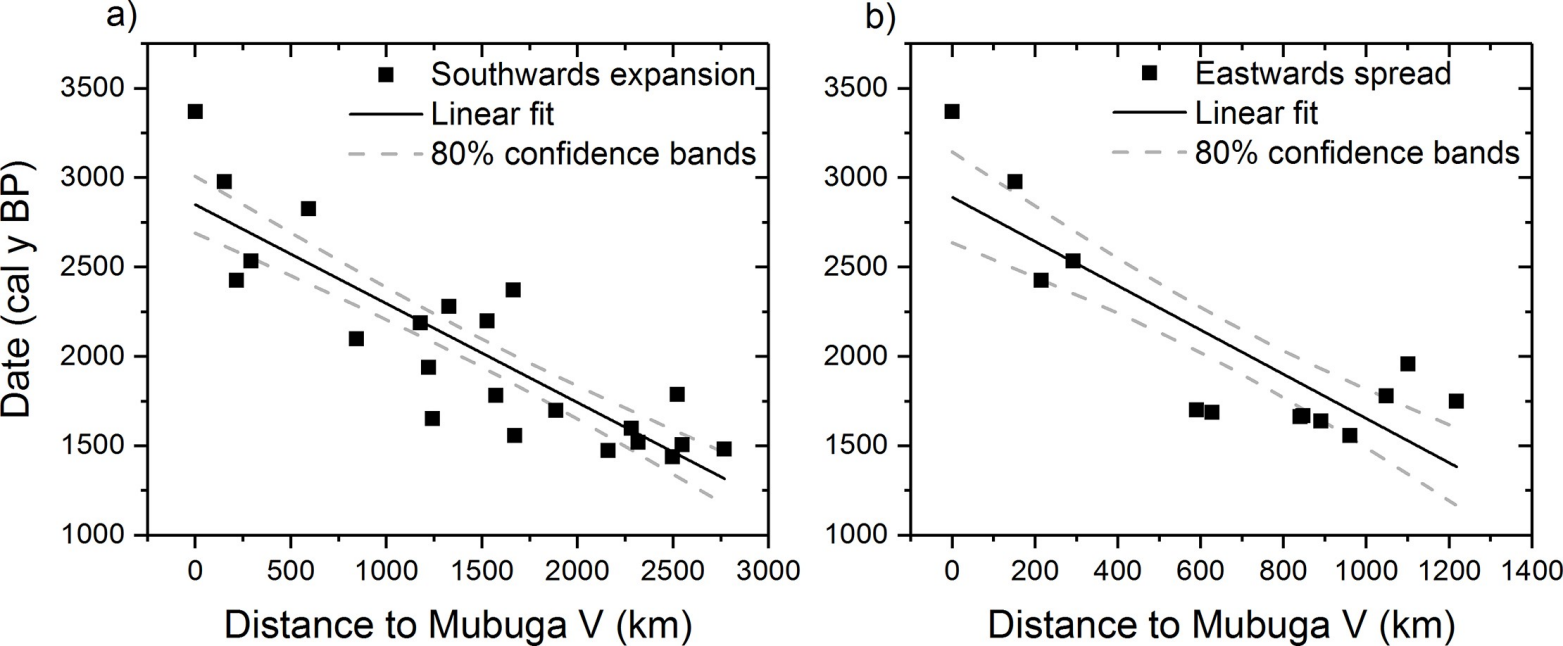
Linear regression of the earliest Bantu data in eastern and southeastern Africa assuming an origin of the spread at Mubuga V. (a) Sites corresponding to a southwards spread from west of Lake Victoria. (b) Sites corresponding to an eastwards spread from west of Lake Victoria.

If we now repeat the regression analysis separately for the eastern and southern data groups, and without excluding the younger dates (i.e., using 64 sites), we obtain slightly better results than with our first approach (especially for the eastern spread), but the correlation coefficients are still very low (Table 1, rows 'Southern data' and 'Eastern data'). For this reason, we shall apply the regression analysis again, but only considering the oldest 31 dates in the database (as already done in the previous subsection, see the row 'Oldest data' in Table 1). Then we have 4 sites in the area west of Lake Victoria, 9 sites in the eastern area (so 13 sites for the eastwards spread) and 18 sites in the southern area (so 22 sites for the southwards spread). The correlation coefficients now improve significantly, reaching values above 0.8 (Table 1, rows 'Oldest southern data' and 'Oldest eastern data'). This implies that the data does indeed follow a linear trend (see Fig 2), which will allow us to obtain reliable estimates for the front speed.

#### 2.1 Southwards spread according to the oldest sites

When considering the southwards spread alone, we now obtain high correlation coefficients, close to |*r*| = 0.8, for all five possible origins of the spread (Table 1, row 'Oldest southern data'). However, we obtain slightly better results when assuming the origin of the spread at the west of Lake Victoria and near it (i.e., any of the five origins except Kalambo Falls, see Fig 1), and especially when considering the two oldest origins (|*r*| = 0.87 for Mubuga V and |*r*| = 0.86 for Kabacusi). For both origins, the slope (speed) is very highly significantly different from zero (*p*<10^−6^). These results imply that the spread process probably began with the first arrival of Bantu speaking people in western East Africa. Therefore, we shall estimate the expansion spread from the two best results. When considering that the origin of the spread took place at Mubuga V, the speed range implied from the linear regression (Fig 2A) is *s* = 1.50–2.11 km/y at the 80% CL, and for an origin at Kabacusi it is *s* = 1.60–2.27 km/y at the 80% CL. Therefore, we can estimate that the spread of agriculture in southeastern Africa took place at an average speed of 1.50–2.27 km/y (80% CL). Using the 95% CL range instead, the final conclusions do not change (Section D in S1 Text).

#### 2.2 Eastwards spread according to the oldest sites

For the eastwards spread, the results only yield good correlation coefficients when considering the oldest dates as origins of the spread, namely |*r*| = 0.84 for an origin at Mubuga V and |*r*| = 0.79 for an origin at Kabacusi. Interestingly, assuming an origin at Kalambo Falls, a site that would in principle not correspond to the eastwards spread, also yields better results that the two possible origins west of Lake Victoria with later dates (last two columns in Table 1). Indeed, for an origin at Kalambo Falls, |*r*| = 0.79, which is similar to the best values, quoted above, that correspond to the two oldest origins (|*r*| = 0.84 for Mubuga V and |*r*| = 0.79 for Kabacusi). For both oldest origins, the slope (speed) is highly significantly different from zero because *p*<0.05. Note that these correlations are less statistically significant than those obtained for the southwards spread (previous paragraph). This is as expected, given the fewer number of sites (N = 13 versus N = 22). However, *p*<0.05 still implies that the correlations are highly statistically significant. These results imply that the spread process eastwards began with the first arrival of Bantu people in East Africa. The front speed ranges obtained for the two best results are *s* = 0.59–1.02 km/y, at 80% CL, for an origin at Mubuga V, and *s* = 0.65–1.27 km/y, at 80% CL, for an origin at Kabacusi. This implies an observed range of speed *s* = 0.59–1.27 km/y (80% CL) for the eastward expansion from the Great Lakes region. Using the 95% CL range instead, the final conclusions do not change (Section D in S1 Text).

#### 2.3 Comparison

Comparing the results for the two spread processes, we see that in both cases the best possible origin is located west of Lake Victoria and dated around 1000–1500 cal BC. Nonetheless, the eastward spread seems to have been slower (0.59–1.27 km/y) than the southwards spread (1.50–2.27 km/y). This difference agrees with the hypothesis that the two spreads may have been led by slightly different ethnolinguistic groups [1, 3], but does it imply that the nature of both spreads was different? Below we shall analyze quantitatively if this difference in the front speeds implies a significant difference in the demic or cultural nature of both spreads.

### 3. Demic versus cultural diffusion

Parameter *C* in Eq (1) is the intensity of cultural diffusion. The higher the value of *C*, the faster the spread rate because more hunter-gatherers are incorporated into the farming populations [26]. This yields the intuitive conclusion that the southwards Bantu spread (1.50–2.27 km/y) may have had a higher cultural component, because it was faster than the eastwards bantu spread (0.59–1.27 km/y). To evaluate the cultural effect on both Bantu spreads, we will compare these observed speed ranges to those predicted by the demic-cultural model [26], i.e. by Eq (1) in the present paper.

To find the theoretical speed from the demic-cultural model, we need to assign values to the parameters in Eq (1). The growth rate *a* has been estimated, from ethnographic data of pre-industrial farming populations settling in empty space, to be in the range 0.023≤*a*≤0.033 y^-1^ (80% CL) [46]. This is the range of growth rates that we shall use here. The growth rate has also been estimated from archaeological data of early farmers in Europe to be 0.024 y^-1^ [47], which lies within the previous range. Another parameter related to population growth is the generation time *T*, defined as the mean age difference between a parent and one of his/her children (not necessarily the first one) [48]. For preindustrial farmers, the generation time has been estimated to be in the range 23≤*T*≤35 y [36, 48], and this is the range we shall use here. Note that, from a reproductive point of view, the combination of the lowest generation time and the highest growth rate yields the fastest population growth and, in consequence, the fastest rate of spread according to Eq (1). Similarly, the lowest reproduction rate and largest generation time will yield the slowest population growth and the slowest spread rate.

The parameters related to the population dispersal, *p*_*i*_ and *r*_*i*_, can be obtained from mobility data of preindustrial farming populations. In particular, we will use information on the distance between the birthplace and the place of residence reported for the Majangir people in Ethiopia [49]. Their area of residence lies slightly further north from the area of spread of the Bantu population, but they are a preindustrial society practicing cereal-based agriculture in a similar environment to the areas where the Bantu population spread. We mention that quantitative mobility data of preindustrial farmers are very difficult to find. For some other African populations there are mobility data, but they are not appropriate for our purposes (see Section C3 in S1 Text). The data reported for the Majangir people correspond to two populations, Shiri and Gilishi, but only the data on three age groups are reliable: Gilishi 10–19 y, Gilishi 20–29 y, and Shiri 10–19 y [49, 50]. Because the age group closer to the generation time *T* range (23–35 y, see above) is Gilishi 20–29 y, this is the population group we shall use in the results presented here, but we include the results from the other populations in S1 Text, Section C. For the Gilishi 20–29 y subpopulation, the dispersal probabilities and distances are respectively *p*_*i*_ = {0.40; 0.17; 0.17; 0.26} and *r*_*i*_ = {2.4; 14.5; 36.2; 60.4} km [46, 49, 50].

The last parameter in Eq (1) is the intensity of cultural transmission *C*. It does not seem possible to perform direct, reliable estimations of this parameter from ethnographic or archaeological data, so we will find the range of *C* that is consistent with each observed front speed range.

#### 3.1 Southwards spread

**Fig 3:**
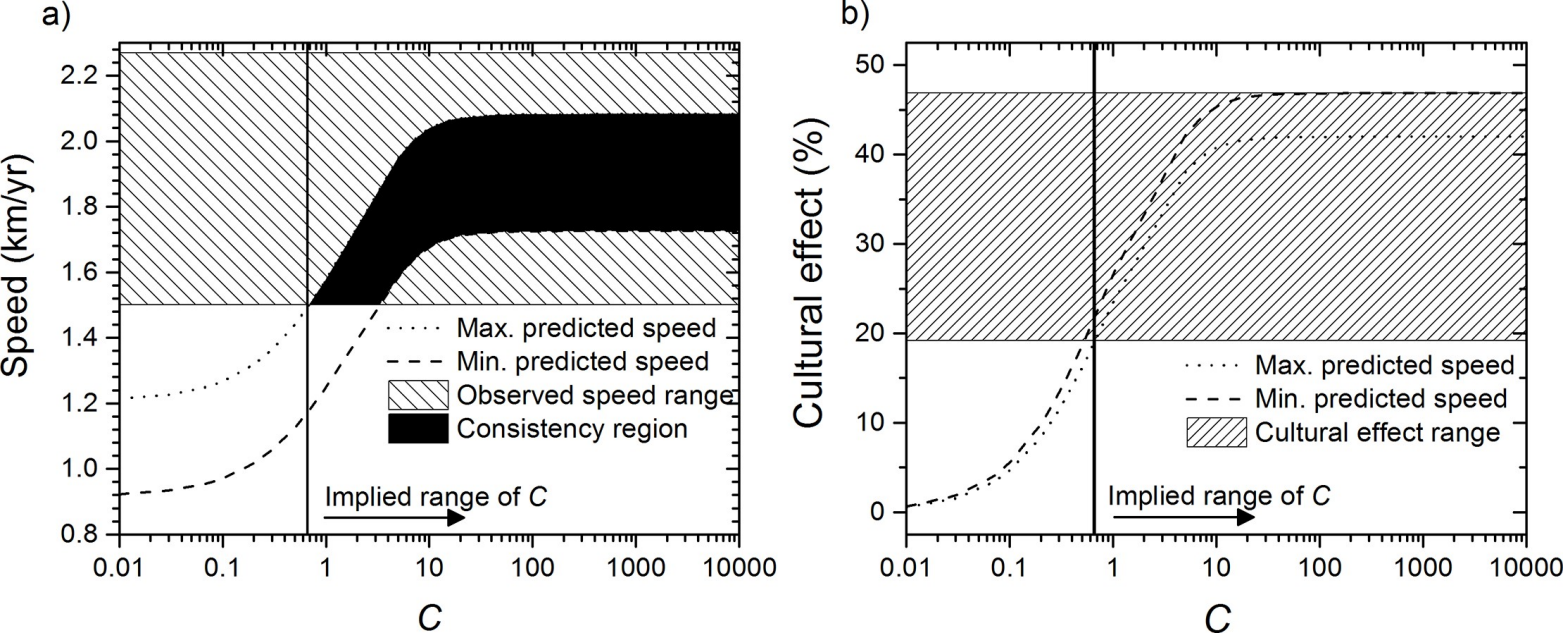
Estimation of the percentage of cultural effect in the southern spread of eastern Bantu. (a) Comparison of the range of the observed speed (hatched rectangle) and that predicted from a demic-cultural model, Eq (1) (area between the dotted and dashed curves). From the consistency region (black area), it follows that *C*≥0.65. (b) Cultural effect predicted by Eq (2) for the maximum and minimum theoretical speeds in panel (a) (dotted and dashed lines). From the range of *C* obtained in (a), i.e. *C*≥0.65, using Fig (b) we reach the conclusion that the cultural effect was in the range 19–47%.

Analyzing first the southwards spread, in Fig 3A we have represented the maximum and minimum front speeds predicted by Eq (1) for the parameter values listed above, as a function of *C*. In Fig 3 we have hatched the range of the observed speed for the Southern spread (obtained in the previous section), i.e. 1.50–2.27 km/y (80% CL). The area shaded in black is the consistency region between predicted and observed speeds, which implies that the intensity of cultural diffusion was *C*≥0.65 for the southwards expansion. From the demic-cultural model, it is possible to estimate the percentage of the cultural effect corresponding to each predicted speed (Eq (2)). In Fig 3B we show the cultural effect as a function of *C*. Using the range *C*≥0.65 estimated above, Fig 3B implies that the percentage of cultural effect involved in the southwards spread was 33 ± 14% (19–47%). This conclusion means that the most important process in the southward Bantu expansion was indeed demic diffusion. Nonetheless, because the upper bound of this range is close to 50%, this yields the possibility that cultural diffusion in the southwards expansion may have played a role nearly as important of demic diffusion. Applying the same analysis using the mobility data for the other two Majangir populations also yields the conclusion that the importance of cultural effect was lower than 50% (see Section C in S1 Text). Finally, we mention that the saturation of the speed for *C*→∞ (Fig 3A) is a general property of reaction-dispersal cohabitation equations, for which the speed for *C*→∞ is equal to the maximum dispersal distance divided by the generation time [26], which is intuitively reasonable (see also Section C4 in S1 Text). On the other hand, the result *C*≥0.65 indicates that more than 65 hunter-gatherers would have been incorporated each generation into the farming communities per every 100 pioneering farmers, which also implies a strong role for cultural diffusion.

#### 3.2 Eastwards spread

**Fig 4:**
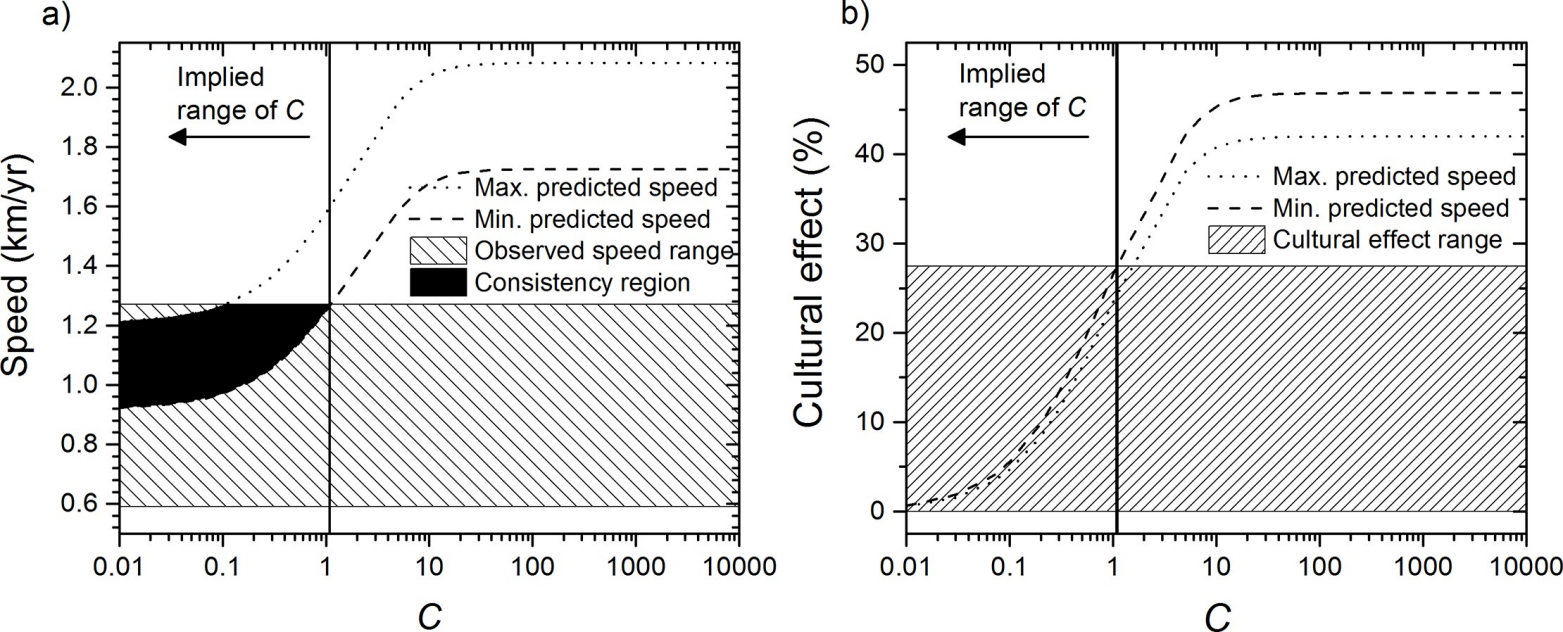
Estimation of the percentage of cultural effect in the eastwards spread of eastern Bantu. (a) Comparison of the range of observed speeds (hatched rectangle) and that predicted from a demic-cultural model, Eq (1) (area between the dotted and dashed curves). From the consistency region (black area), it follows that *C*≤1.1. (b) Cultural effect predicted by Eq (2) for the maximum and minimum theoretical speeds in panel (a) (dotted and dashed lines). From the range of *C* obtained in (a), i.e. *C*≤1.1, using panel we reach the conclusion that the cultural effect was in the range 0–28%.

In Fig 4 we have plotted anew the range of theoretical speeds predicted by Eq (1), but now the hatched area corresponds to the observed speed for the eastwards spread (obtained in the previous section), i.e. 0.59–1.27 km/y (80% CL). We see that, in contrast to the southwards spread, which implies a lower bound for *C* (Fig 3A), for the eastwards spread the observed speed implies an upper bound for *C*, namely *C*≤1.1 (Fig 4A). Reasoning as above, from this range we estimate in Fig 4B the importance of the cultural effect in the Bantu spread into East Africa to be around 14±14% (0–28%). Again this would imply that demic diffusion was indeed the main driving process of the eastwards spread, with possibly very low cultural diffusion involved. Similarly, when using the dispersal data from the other two Majangir populations we obtain that the cultural effect was below 30% (see section C in S1 Text). On the other hand, the result *C*≤1.1 indicates that less than 11 hunter-gatherers would have been incorporated each generation into the farming communities per every 10 pioneering farmers.

#### 3.3 Comparison

Alternatively to the 80% CL ranges for the speed used in the previous subsections, we could focus on the best fits (i.e., the mean speeds obtained above using Mubuga V and Kabacusi as plausible origins). Then the speed for the southwards spread is 1.81–1.94 km/y, which according to Fig 3A implies that *C*≥3 and Fig 3B yields a cultural effect of 33–47%, and the speed for the eastwards spread is 0.81–0.96 km/y, which according to Fig 4 implies that *C*≤0.08 and a cultural effect of only 0–4%. Note that this range *C*≤0.08 suggests a very weak role for cultural transmission, as less than 8 hunter-gatherers would have been incorporated each generation into the farming communities per every 100 pioneering farmers in the eastwards spread.

Either using the 80% CL ranges or the best fits, comparing the results from the two populations we see that they all imply that the spread of agriculture and Bantu languages into southeastern Africa was mainly demic, in agreement with most archeological, linguistic and genetic studies [2, 4, 7, 10, 12, 17, 21, 22]. Nonetheless, we see that the cultural effect seems to have been more important in the southwards spread than in the eastwards spread. This would agree with the observations that, in the eastwards spread, the Bantu people did not initially absorb the local population [1], whereas for the southwards spread, genetic and linguistic studies have implied a higher degree incorporation of local populations, especially Khoikhoi herding populations at the south [3, 23]. Indeed, a very clear impact of the Khoikhoi appears in southern Bantu herding vocabulary, where the words for sheep, cattle and milk came from the local Khoikhoi language. It should be noted, however, that this interaction could have taken place after the spread of the Bantu wave of advance (see Ref. [3], pp. 228–229). Similarly, a strong genetic singularity has been detected in present southern Mozambique, and it has been suggested that this may reflect a strong assimilation of non-Bantu populations in the South [23]. However, ancient genetic data are missing, and our results do not provide conclusive support to this hypothesis of stronger cultural diffusion southwards than eastwards, because the ranges for the importance of the cultural effect in the spread in each direction overlap in the range 19–28%. Therefore, this could also imply that agriculture spread in all of southeastern Africa demically with relatively low cultural input. On the other hand, the observed differences in the rate of spread could be due, alternatively, to geographic or environmental differences rather than to a sociocultural effect. For example, perhaps the differing speeds Eastwards and Southwards could be due to different dispersal kernels in both directions (values of *p*_*i*_ and *r*_*i*_). Unfortunately, at present it is not possible to test this possibility, because we have only ethnographic data for the dispersal kernel. However, it has been proposed that ancient genetics could be used in the future to measure the dispersal kernels of prehistoric populations [28, 51] and, therefore, to test this possibility. In fact, we have not used an heterogeneous environment precisely because of the absence of data to estimate different dispersal kernels, growth rates and/or generation times for different regions. The main point is that demic diffusion was more important than cultural diffusion also for other dispersal kernels reported in ethnographic observations (see Section C in S1 Text).

The model applied in the present paper was developed in Ref. [26]. This model is simple enough so that we can estimate all demic parameters (from independent data), the cultural transmission intensity *C* (from the spread rate), and the cultural effect. However, it is certainly possible to extend this model into more complex ones (including, e.g., a kernel for cultural diffusion [28], age-dependent demic [52] and/or cultural dependencies, etc.). Such models will have more parameters and, therefore, additional uncertainties, so it is reasonable to begin with the simplest possible model, as done in Ref. [26] and the present paper. However, in future work it would be interesting to analyze the effect on the results of using more complex models.

## Conclusions

The spread of agriculture in most of southern Africa is often linked to the spread of Bantu languages, which from an origin in central Cameroon expanded southwards and eastwards, reaching the coast of East Africa and as far south as South Africa. Many authors have tried to elucidate the paths and nature of the spread into and/or around the rainforest area [4, 5, 12]. In contrast, here we have attempted to estimate the spread rates and asses their demic or cultural nature. We have focused on the area on which there is more agreement on the spread paths, namely the eastern half of subequatorial Africa, east of the rainforest. Agriculture in this area was characterized by the cultivation of cereals, unlike previous Bantu populations, and it is apparently upon reaching eastern Africa that the Bantu people adopted metallurgy [1, 3]. Therefore, this stage of the Bantu spread is singular enough to be studied separately from previous spreads.

Applying linear regression to the earliest Bantu data, we have observed that statistically sound estimations of the spread rates are achieved when considering two streams, one eastwards from west of Lake Victoria (which could correspond to the Kaskazi ethnolinguistic group according to Ehert [4]) and the other one southwards (which could correspond to the Kusi ethnolinguistic group [4]). For both directions, our results indicate that the spread began as soon as the first Bantu people reached the area west of Lake Victoria, by the beginning of the last millennium BC [4].

An unexpected result of this work is that the speeds obtained from the regression analyses (0.59–2.27 km/y) are substantially slower than previous estimations (9–15 km/y), which were based on few data [2, 45]. Moreover, we have shown that the eastern spread into East Africa was clearly slower (0.59–1.27 km/y, 80% CL) than the southwards spread (1.50–2.27 km/y, 80% CL). When comparing these observed ranges to the speeds predicted from a demic-cultural model [26], we reach the conclusion that, in spite of the speed differences, demic spread was the main driver in both directions. Nonetheless, the results show that the cultural effect was probably stronger in the southern expansion (19–47%) than in the eastwards expansion (0–28%). This could agree with the hypothesis from herding loanwords [3] and genetic data [23] that, in the southern spread, Bantu populations incorporated people from local populations, especially Khoikhoi pastoralists in the southern areas. Unfortunately the results obtained are not conclusive in this regard. In the future, when more accurate and complete archaeological databases become available, the methodology explained in the present paper may lead to more conclusive results on this specific issue (additionally, the methods in Ref. [27] could be applied to ancient genetic clines, if they are detected in the future).

Finally, it is interesting to note that, contrary to the conclusions reached for the spread of Khoikhoi populations in the southwest of Africa (outside of the area considered here), where the spread *of herding* was faster (1.4–3.3 km/y, also with 80% CL) and apparently driven mainly by cultural diffusion [29], we have observed that the spread of Bantu *agriculture* was mostly demic. This conclusion agrees and reinforces the hypothesis, postulated previously [29], according to which when animal and plant domestication spread in the form of *farming* economics, the driving mechanism is demic diffusion (as observed here,in Europe [26, 51], eastern and southeastern Asia [53]), whereas *herding* might be easier to learn, allowing the main driving mechanism to be cultural diffusion in the spread of pastoralism (as observed in southwestern Africa [29]). Interestingly, this hypothesis (i.e., mainly demic diffusion for farming versus mainly cultural diffusion for herding) agrees with the work by Sørensen, who has argued that herding is substantially easier and faster to adopt by hunter-gatherers than farming [54]. Moreover, for some hunter-gatherer populations it has been reported that some individuals tried to establish themselves on an agricultural basis (after learning the techniques by assisting their agricultural neighbors), but all of them failed. In contrast, some of those same hunter-gatherers successfully became herders [55]. Again, this suggests that it is rather difficult for hunter-gatherers to farm on their own with the same success than the farmers who have taught them, and that this difficulty is less severe in the case of herding. Therefore, there is ethnographic support for the proposal that herding could be a simpler cultural trait to transmit than farming, and that this difference could explain the faster Neolithic spread rates of herding [29] as compared to those of farming (Refs. [26, 51, 53] and the present paper).

## Supporting information

S1 Text Supplementary methods and results. Database selection, effect of late Bantu dates, results for other dispersal kernels, and effect of the confidence level for the observed speed range.
(DOCX)

S1 Data Early Iron Age (Bantu) sites in East and Southeast Africa. Names, dates, coordinates, spread periods, spread areas, distances to origins and sample types (materials dated).
(XLSX)
