# Supplementary material for: Assessing the importance of cultural diffusion in the Bantu spread into southeastern Africa
Source: PLoS One. 2019 May 8;14(5):e0215573. doi: 10.1371/journal.pone.0215573 (PMC6506142; doi:10.1371/journal.pone.0215573)
Supplement: S1 Text — Database selection, effect of late Bantu dates, results for other dispersal kernels, and effect of the confidence level for the observed speed range. (DOCX) [file pone.0215573.s001.docx]

N. Isern and J. Fort, Assessing the importance of cultural diffusion in the Bantu spread into southeastern Africa

# S1 Text. Supplementary methods and results

## A. Database selection

From the 107 Early Iron Age (EIA) dates published by Russell et al. (table S2 in Ref. [1]), we have selected the 71 dates that fall in the area which corresponds to the eastern spread according to the literature (although some areas may have also had influences from a western Bantu spread) [2-4]. From these eastern dates, we have rejected that for Taukome because its coordinates locate the site under the sea, and hundreds of kilometers away from a site called Taukome in Botswana [3]. Thus below we consider 70 dates (included as S1 Data).

The current chronology indicates that Bantu populations and the EIA reached the west of the Great Lakes area by the beginning of the last millenium BC, and that they did not start spreading outside of the intralacustrine area until the second half of the last millennium BC [5-7]. Therefore, dates prior or around 1000 BC will be suspect of corresponding to pre-Bantu cultures, and the same applies to substantially earlier dates than their neighbors. Below we discuss the dates that we have rejected as corresponding to pre-Bantu cultures, as well as those which we consider dubious. The dates that we have rejected are represented as squares in Fig A below.

### Excluded dates

Here we discuss the 6 dates that we have excluded, and the reasons for doing so.

The database contains a very early date for Kwelikwiji (1397 cal BC) in eastern Tanzania. However, this date was already considered at the moment of its dating as being the result of either a disturbance, an old wood effect, or indicative that the dated pottery belongs to a pre-EIA culture [8]. For this reason, we shall not consider this date in our analysis.

In Zambia we find two additional doubtful dates: Nakapapula (1570 cal BC) in central Zambia and Kumadzulo (1187 cal BC) in southern Zambia. Both sites are dated considerably earlier than the arrival times assumed from archaeological data [5, 9]. In addition, the date in Nakapapula has been previously classified as corresponding to the Late Stone Age, with no evidence of metallurgy, and with the first evidences of pottery appearing considerably later [9]. The date for Kumadzulo was marked as “rejected as invalid, usually by the excavator” in earlier publications by Derricourt [9], so we shall follow this assessment and reject this date. Therefore, we shall not include neither of the two dates in our analysis. In the same region, the date for Ndonde (420 cal BC) was considered as contaminated by disturbance of the subsoil, and thus unreliable [10]. So we reject this date.


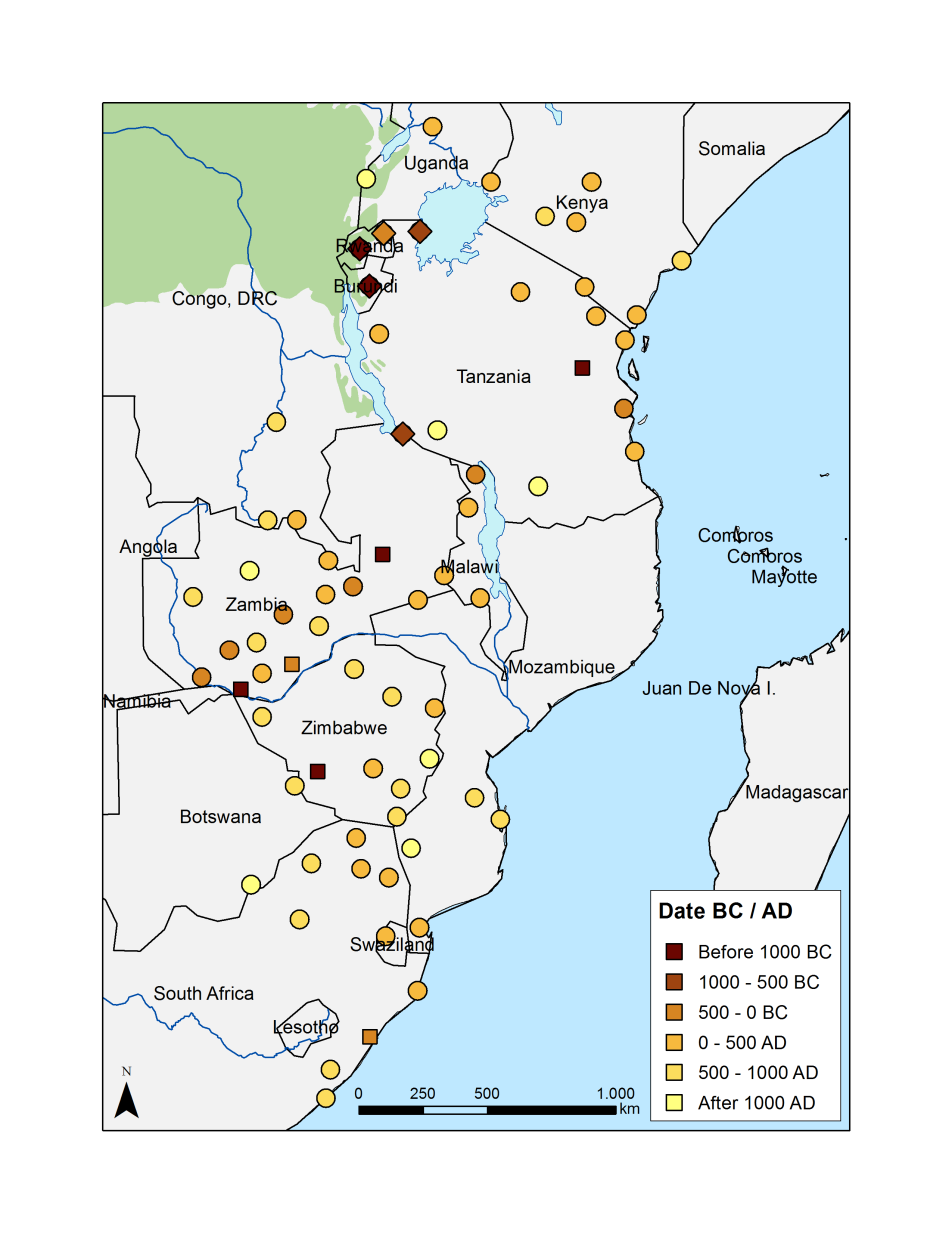


**Fig A**. Eastern and southeastern Bantu sites. Circles indicate Bantu sites included in the analysis. Diamonds stand for very early, likely Bantu sites. Squares are likely pre-Bantu sites excluded from the analysis. All symbols are colored according to their calibrated date.

In southern Zimbabwe, the date from the Bambata Cave Series (1027 cal BC) corresponds to findings of Bambata pottery [11], a few centuries earlier than the expected arrival of the Bantu populations and the EIA [5, 11]. Although the introduction of pottery is often related to the Bantu spread, the Bambata pottery is often assumed to have been introduced by hunter-gatherer populations [12], or Ehret has also hypothesized a possible East Sahelian origin from a pre-Bantu southward expansion [5]. In any case, though, this date would correspond to a period previous to the Bantu expansion, so we do not include it in our analysis.

Finally, the site Shongweni Waterworks Park (44 cal BC) in South Africa is dated considerable earlier than the assumed arrival time at about 400 cal AD [5]. In addition, the date was obtained from charcoal found between the LSA and EIA layers at the site, in which the EIA occupation is dated from about 850 cal AD [11]. For this reason we shall not include this site in our analysis.

## B. Effect of late Bantu dates

In the main paper we want to estimate the average rate at which agriculture and the Bantu language spread into eastern and southeastern Africa. However, when applying linear regression to the archeological dates versus their distances to the oldest sites, we obtain very poor correlation coefficients. In Fig B, panel A we show the regression obtained when using Mubuga V as possible origin of the spread. We see that, for a given distance from the origin, the plot includes very early dates but also very late ones (for example, at 1000 km we find dates on a span of 2000 years), and therefore they cannot all correspond to the earliest Bantu spread. Thus we have taken out of the analysis the dates that clearly correspond to later processes. First of all, since the Bantu range expansion is assumed to have reached South Africa by 400 AD, we take out of the analysis all dates later than 600 cal AD (1350 cal BP). We have then assessed the remaining dates and taken out of the analysis those which are clearly later than the surrounding sites. Fig C shows the database (a) before and (b) after this filtering process, and the interpolation map obtained from each group of dates. The interpolation of the filtered database shows substantially less noise, and the progression of the Bantu front is more obvious (Fig C, panel b). If we now apply regression analysis with the filtered database (Fig B, panel b), we see that the data now follows a more linear trend, in agreement with the fact that the correlation coefficient is now larger, namely |*r*|=0.65.


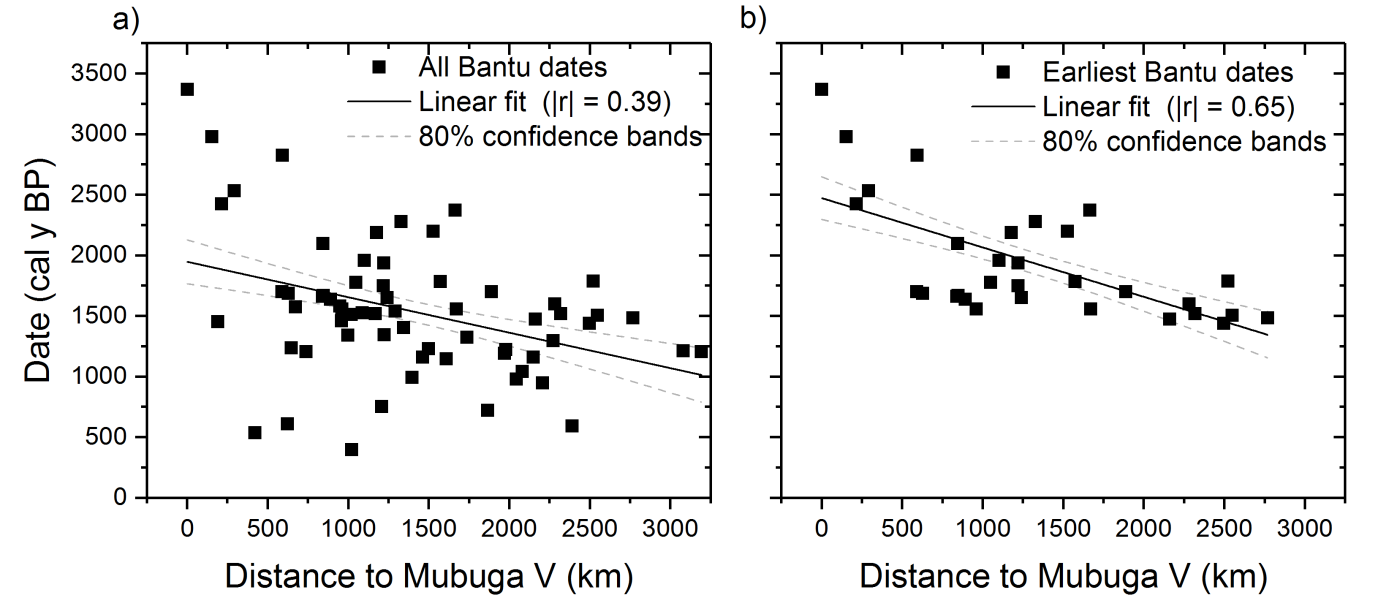


**Fig B**. Linear fit of the Bantu dates with an assumed origin at Mubuga V. (a) contains all dates in S1 Data. (b) contains only the dates classified as “Early Spread” in S1 Data. The correlation coefficients |*r*| that appear in these Figs are also included in the main paper, Table 1, rows 'All data' and 'Oldest data'.


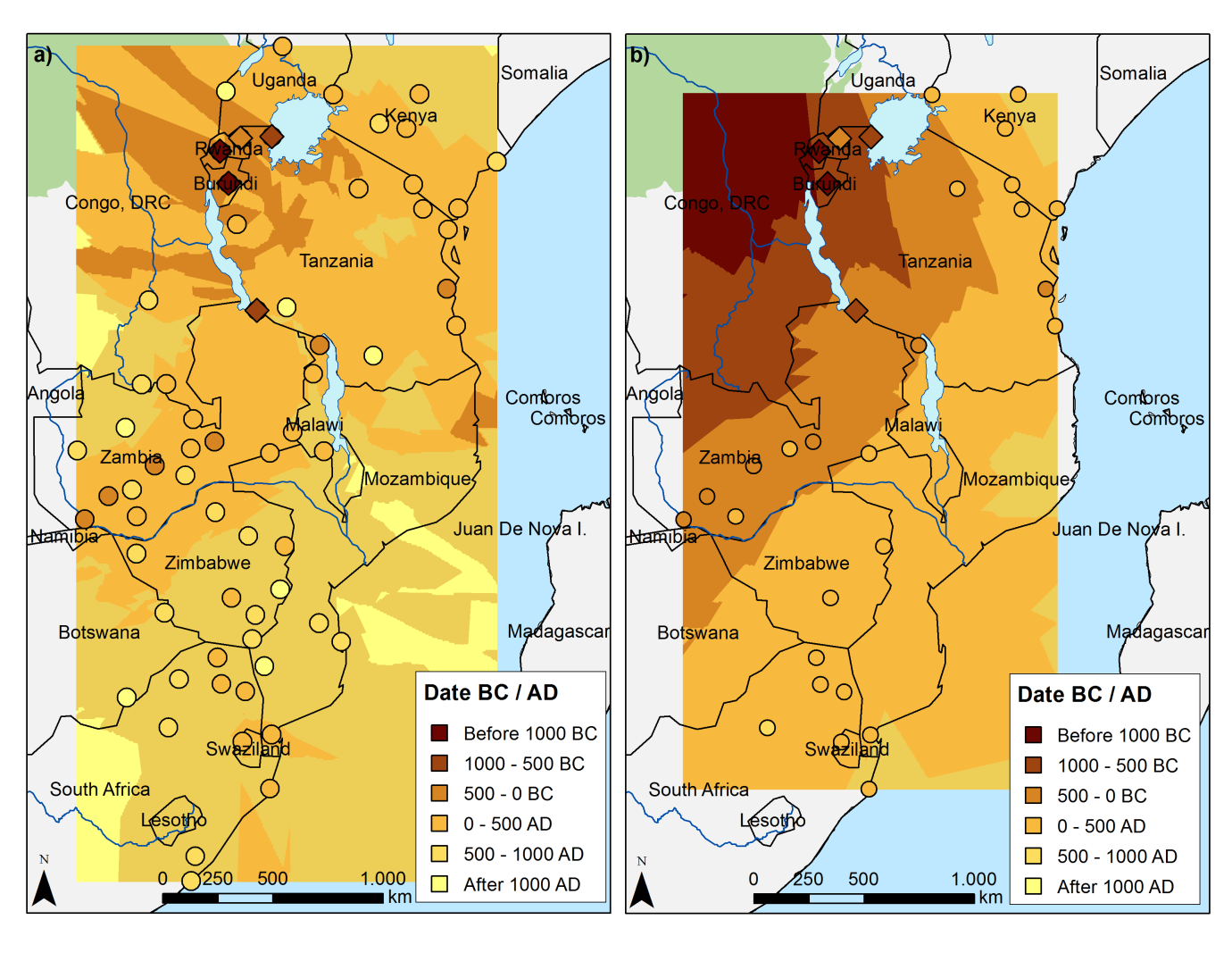


**Fig C**. Database and interpolation map of arrival times of agriculture. (a) Contains all dates in S1 Data. (b) Contains only the dates classified as “Early Spread” in S1 Data. Interpolations have been performed using universal linear kriging with the ArcGIS Spatial Analyst extension.

## C. Results for other dispersal kernels

Stauder [13] collected dispersal data for two Majangir communities, Shiri and Gilishi, who practice cereal cultivation in the fringes of the savanna in Ethiopia. Each dispersal kernel refers to distances from the place of birth to the place of residence of individuals. Such distances are more appropriate to study the propagation of populations fronts than, e.g., distances between birthplaces of mates. However, only the data on three of the age subgroups are reliable: Shiri 10-19 y, Gilishi 10-19 y and Gilishi 20-29 y [15]. Because the generation time for preindustrial agriculturalist populations has been estimated to be in the range $29\leq T\leq35$ [14], in the main paper we have used the data for Gilishi 20-29 y. In Secs. C1 and C2 below, we include the results obtained for the other two groups. Useful dispersal data are very difficult to find. In Sec. C3 below, we discuss why the available dispersal data for other African populations are not appropriate for our purposes.

### C1. Gilishi 10-19 y

The dispersal probabilities and distances for this population are respectively $p_{i}=\left\{ 0.54;0.17;0.04;0.25 \right\}$ and $r_{i}=\left\{ 2.4;14.5;36.2;60.4 \right\}$ km [13, 15, 16]. Figs D and E are equivalent to Figs 3 and 4 in the main paper, but using this dispersal kernel. The results imply that the importance of the cultural effect was 36.2±13.3% (22.9-49.5%) for the southern spread and 13.5±13.5% (0-27%) for the eastern spread. These results are very similar to the ones reached in the main paper, thus the conclusions do not change.


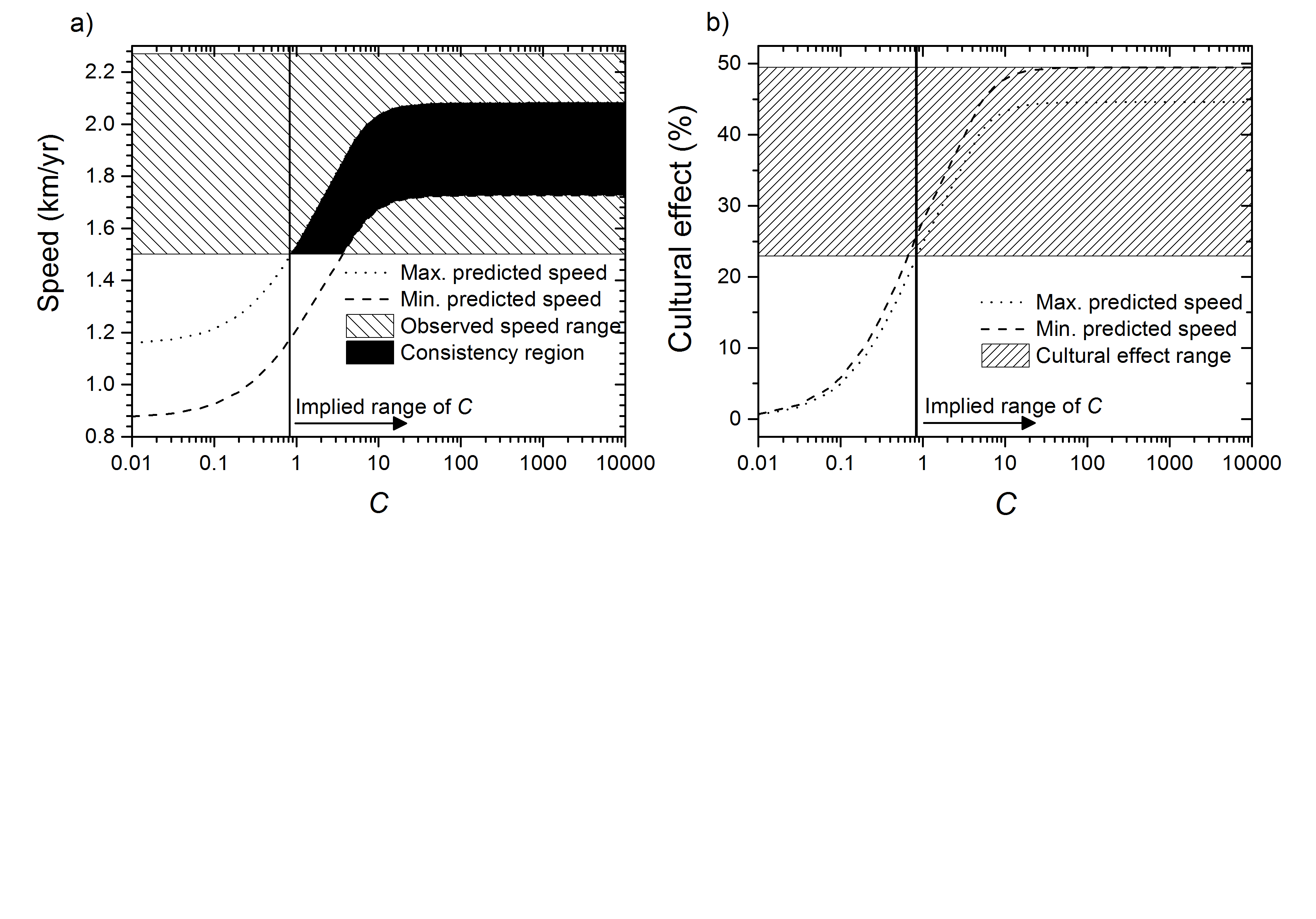
**Fig D**. Analysis of the southern spread of eastern Bantu using the dispersal data from ‘Gilishi 10-19 y’. (a) Comparison of the 80% CL range of observed speeds (hatched rectangle) and that predicted from a demic-cultural model, Eq. (1) in the main paper (area between the dotted and dashed curves). From the consistency region (black area), it follows that $C\geq0.83$. (b) Cultural effect predicted by Eq. (2) for the maximum and minimum theoretical speed in panel (a) (dotted and dashed curves). From the range of *C* obtained in (a), i.e. $C\geq0.83,$using Fig (b) we reach the conclusion that the cultural effect was in the range 22.9-49.5%.


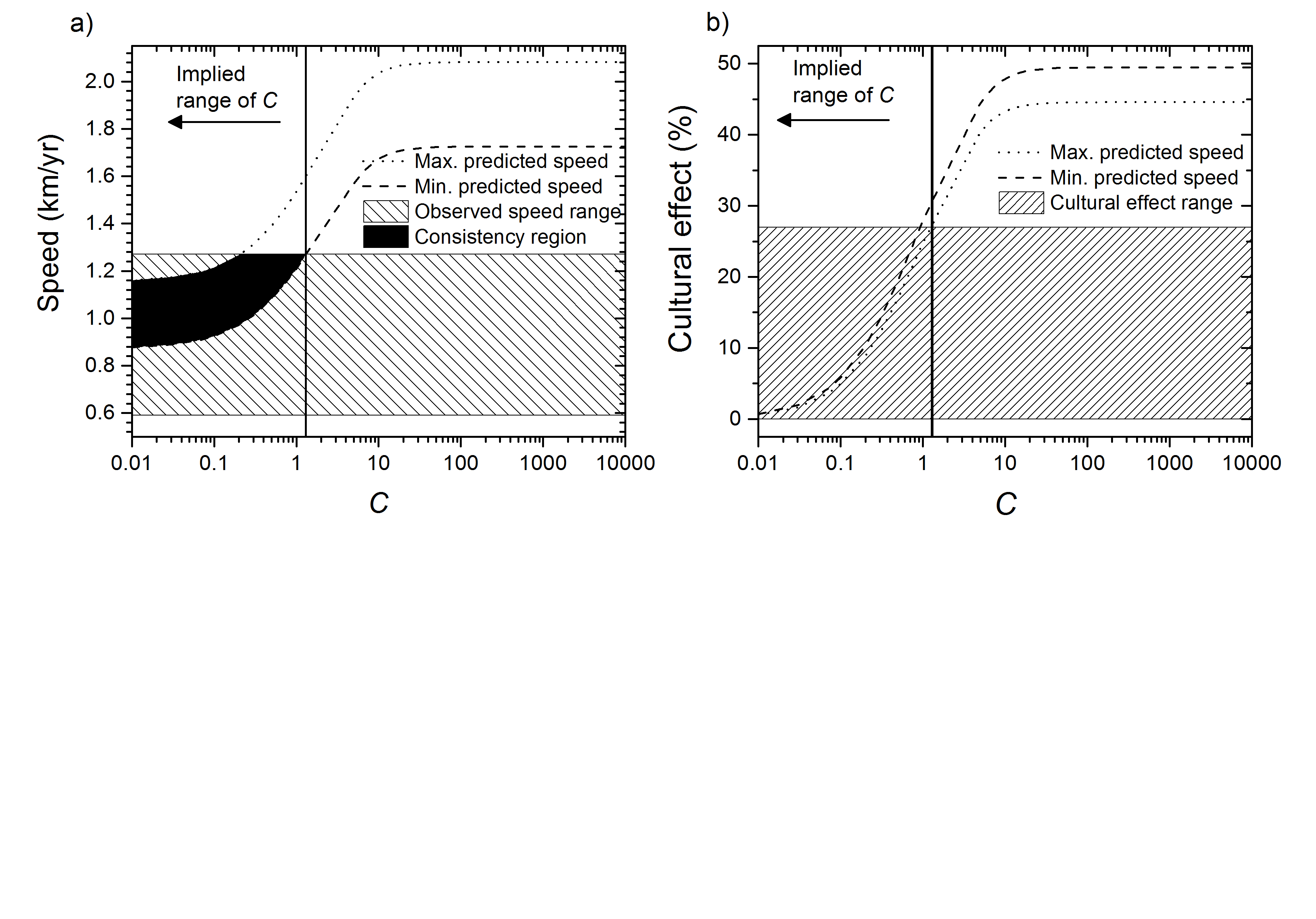
**Fig E**. Analysis of the eastwards spread of eastern Bantu using the dispersal data from ‘Gilishi 10-19 y’. (a) Comparison of the 80% CL range of observed speeds (hatched rectangle) and that predicted from a demic-cultural model, Eq. (1) in the main paper (area between the dotted and dashed curves). From the consistency region (black area), it follows that $C\leq1.3$. (b) Cultural effect predicted by Eq. (2) for the maximum and minimum theoretical speed in panel (a) (dotted and dashed curves). From the range of *C* obtained in (a), i.e. $C\leq1.3,$ using Fig (b) we reach the conclusion that the cultural effect was in the range 0-27%.

### C2. Shiri 10-19 y

The dispersal probabilities and distances for this population are respectively $p_{i}=\left\{ 0.19;0.17;0.22;0.52 \right\}$ and $r_{i}=\left\{ 2.4;14.5;36.2;60.4 \right\}$ km [13, 15, 16]. Figs F and G are equivalent to Figs 3 and 4 in the main paper, but using this dispersal kernel. The results imply that the importance of the cultural effect was 24.2±22.7% (1.5-46.9%) for the southern spread and 6.1±6.1% (0-12.2%) for the eastern spread. These results yield a wider range for the cultural effect in the southern spread, but it still implies that in both directions the spread was mostly demic (cultural effect below 50%). The two ranges again overlap. Therefore, all of the conclusions in the main paper remain unchanged.


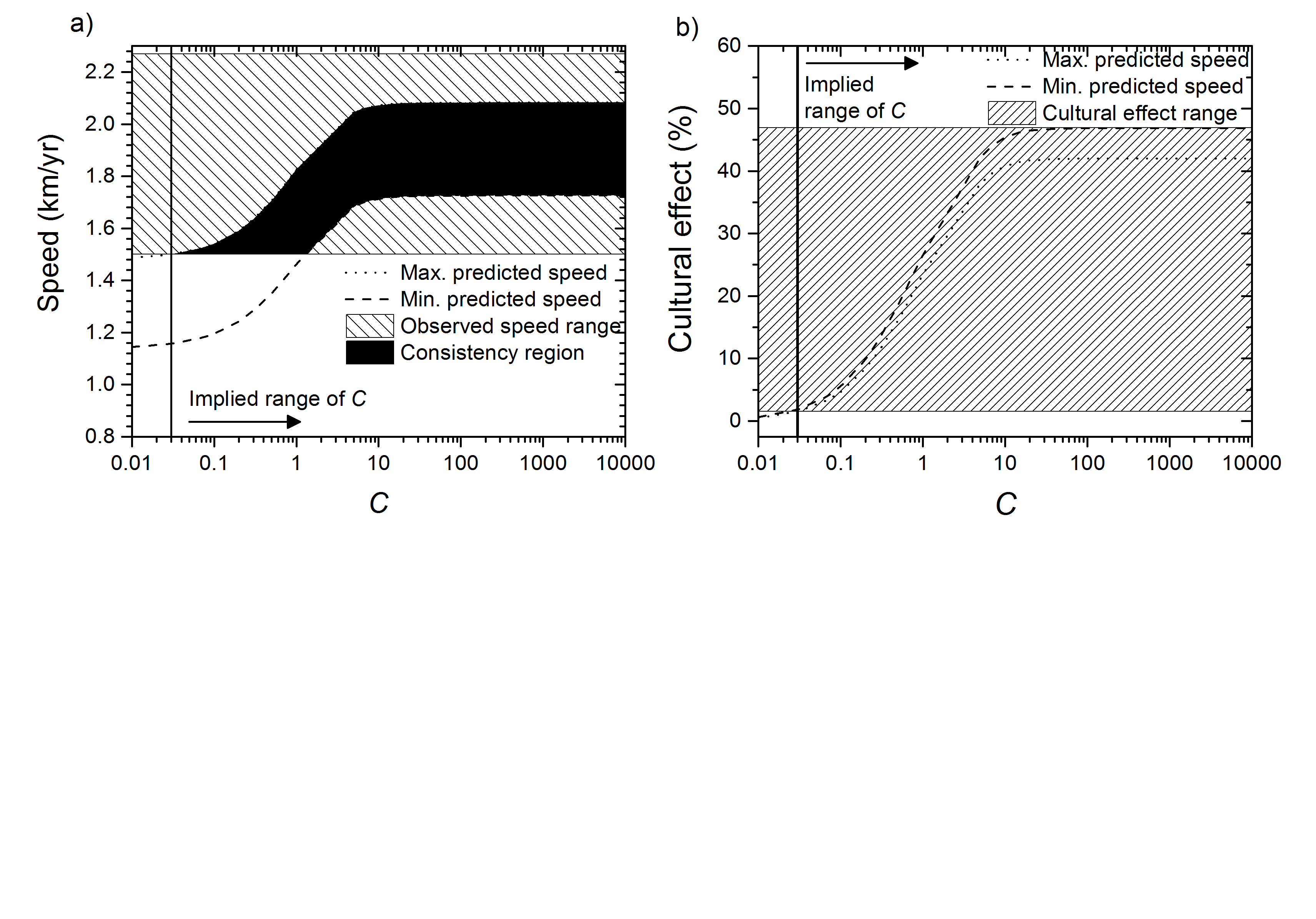
**Fig F**. Analysis of the southern spread of eastern Bantu using the dispersal data from ‘Shiri 10-19 y’. (a) Comparison of the 80% CL range of observed speeds (hatched rectangle) and that predicted from a demic-cultural model, Eq. (1) in the main paper (area between the dotted and dashed curves). From the consistency region (black area), it follows that $C\geq0.03$. (b) Cultural effect predicted by Eq. (2) for the maximum and minimum theoretical speed in panel (a) (dotted and dashed curves). From the range of *C* obtained in (a), i.e. $C\geq0.03,$ using Fig (b) we reach the conclusion that the cultural effect was in the range 1.5-46.9%.


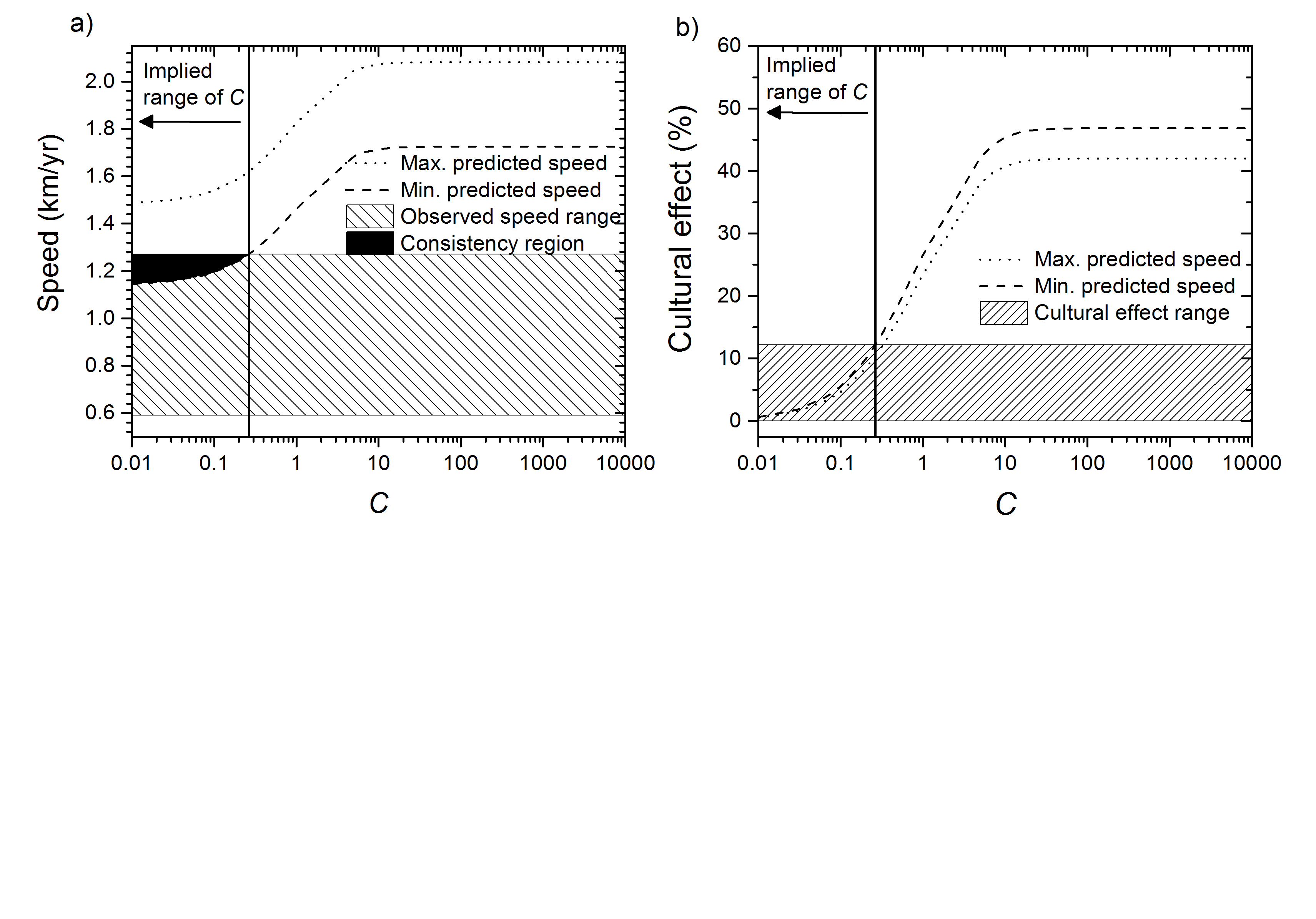
**Fig G**. Analysis of the eastwards spread of eastern Bantu using the dispersal data from ‘Shiri 10-19 y’. (a) Comparison of the 80% CL range of observed speeds (hatched rectangle) and that predicted from a demic-cultural model, Eq. (1) in the main paper (area between the dotted and dashed curves). From the consistency region (black area), it follows that $C\leq0.26$. (b) Cultural effect predicted by Eq. (2) for the maximum and minimum theoretical speed in panel (a) (dotted and dashed curves). From the range of *C* obtained in (a), i.e. $C\leq0.26,$ using Fig (b) we reach the conclusion that the cultural effect was in the range 0-12.2%.

### C3. Other dispersal data

While preparing this work, we have searched the literature for other dispersal data of African agricultural populations, but none seemed adequate or reliable enough. Below we discuss several of the dispersal data available on the literature.

One dataset from Africa corresponds to distances between the birthplaces of husbands and wives for the Issongos [16, 17], a Bantu-speaking tribe in the Central African Republic [18]. Cavalli-Sforza recorded these data for the Issongos and also for Babinga pygmies [15, 17], who are Aka hunter-gatherers that live in a symbiotic relationship with the Issongos [18] (see also Ref. [19], pp. 11, 20, and 42-43). However, it has been noted that the Issongos have an anomalously low mobility due to some very specific features, namely that they occupy a very limited area and, moreover, few marriages occur with other tribes [15]. This suggests that their mobility pattern is rather different from that of most cereal cultivators, so we have decided not to apply this dataset.

A second dataset, reported by Watts [20], refers to marriage distances of Yoruba [21] women in and around the city of Ilorin, the capital of Kwara State, Nigeria. According to Watts [20], Ilorin is a very large city (about 400,000 people) with many incentives (employment opportunities, medical care, schools, piped water, etc.) that attracts people from surrounding areas. This causes much more migration from nearby small villages to Ilorin than vice versa. Watts [20] considers that this reflects the dependence of rural, poor settlements on the city. Clearly, this situation is very different from those faced by the populations involved in the Bantu expansion, which did not have large cities and were unaffected by similar incentives to migrate to specific locations. Obviously, these differences are substantial and may lead to very different distributions of marriage distances, so it does not seem reasonable to apply this dataset for our purposes. Moreover Watts [20] considered only Okelele, a section of the city of Ilorin for which marriage distances are substantially lower as compared to the rest of the city, so this database is representative only of a subsample of the population.

A third dataset refers to marriage distances for four villages in Chad of the Sara Madjingay [22], agriculturalists who cultivate millet and sorghum in addition to several root plants [23] (very similarly to the Bantu people spreading in southeastern Africa [24, 25]). Unfortunately, this dispersal kernel is incomplete because it does not include distances for individuals born outside the studied area (neither distances for individuals who migrated away from this area) [22]. Such larger distances may have an important effect on the predicted speeds, so this dataset is not appropriate for our purposes.

### C4. Discussion

It is worth to compare some aspects of the three kernels used. According to Eq. (1) in the main paper, the spread rate depends on the kernel, and this is why the curves are different in Figs 3-4 (first kernel), Figs D-E (second kernel) and Figs F-G (third kernel). Note, however, that the saturating spread rate (speed for $C\to\infty$) is the same for all three kernels. This is due to the fact that the speed for $C\to\infty$ is simply the maximum dispersal distance divided by the generation time [26], and the maximum dispersal distance is the same (60.4 km) for all three kernels (because unfortunately we do not have the raw dispersal distance for each individual of the three populations, but only the three histograms reported in Ref. [13]). According to Eq. (2) in the main paper, this saturation of the spread rate implies, in turn, the saturation of the cultural effect (also for $C\to\infty$), which is seen in panels b of Figs 3-4 and D-G. In these Figs, we note that the saturating value of the cultural effect is not exactly the same for the three kernels (this is due to the fact that $s_{demic}$, i.e. the spread rate for $C=0$, is not exactly the same for the three kernels). However, it is interesting to note that the saturating value of the cultural effect is always below 50% (although it is very close to 50% for the maximum speed predicted by the kernel in Figs D-E). Therefore, those three kernels imply the primacy of demic over cultural diffusion (irrespective of the value of the spread rate). However, this will not be necessarily the case for other kernels. For example, a kernel with a maximum distance longer than that for the three kernels used (60.4 km) will surely have faster saturating speeds and (if the demic speed is similar) higher saturating percentages of the cultural effect. Therefore, it is important to continue searching for additional ethnographic data on dispersal kernels of pre-industrial African farmers. Ancient genetic data could be also used for this purpose (see the main paper, Results and discussion, subsection 3.3).

## D. Effect of the confidence level for the observed speed range

In order to compute the observed speed range (from the regression slopes and their errors), in the main paper we have used a 80% confidence level (CL). This makes direct comparison possible to Ref. [27], where the same method (also with a 80% CL) was applied to analyze a non-Bantu Neolithic spread (namely, that of Khoikhoi herders in southern Africa). In this section we repeat the calculations with a 95% CL, and find that the conclusions do not change.

### D1. Southern spread

In the main paper, Results and discussion, subsection 2.1, we have seen that for the southern spread the linear regression using Mubuga V as origin (Fig 3a) yields the speed range 1.50-2.11 km/yr (80% CL) and the regression using Kabacusi as origin yields 1.60-2.27 km/yr (80% CL). For this reason, in Fig 3a in the main paper we have applied the overall range 1.50-2.27 km/yr (80% CL). Here, instead of a 80% CL, we use a 95% CL. This yields wider ranges, as it should, namely 1.32-2.29 km/yr (95% CL) for Mubuga V and 1.40-2.47 (95% CL) for Kabacusi. Therefore, the overall range is 1.32-2.47 km/yr (95% CL). Fig H, panel a is the same as Fig 3a in the main paper, but using the 95% CL range (1.32-2.47 km/yr) instead of the 80% CL one (1.50-2.27 km/yr). Since the curves are the same as in Fig 3a, the smaller lower bound for the observed speed (1.32 km/yr in Fig H, panel a, compared to 1.50 km/yr in Fig 3a) leads to a lower value for the minimum cultural transmission intensity ($C\geq0.2$ in Fig H, panel a, compared to $C\geq0.65$ in Fig 3a). Applying that $C\geq0.2$ (vertical line) in Fig H, panel b, we obtain the range 8-47% for the cultural effect. As expected, this is wider than the result from Fig 3b (19-47%). In both cases, the maximum cultural effect is the same, i.e. 47%, due to the saturation of the speed in Fig H, panel a. The important point is that the maximum cultural effect (47%) is below 50%. Thus we conclude that the spread southwards was mainly demic, independently of whether we use a 80% CL (Fig 3) or a 95% CL (Fig H).


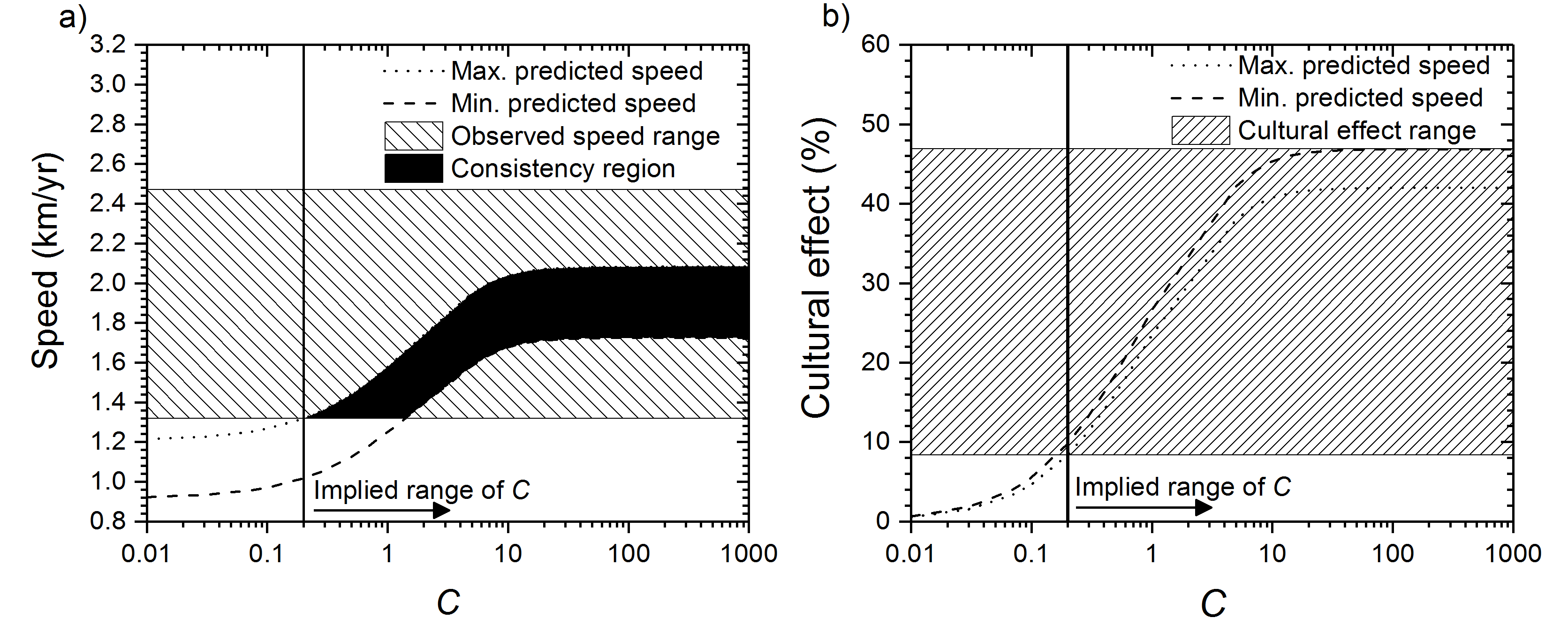


**Fig H**. Southern spread. This Fig is the same as Fig 3 in the main paper, but using a 95% CL for the observed speed range instead of a 80% CL.

### D2. Eastern spread

In the main paper, Results and discussion, subsection 2.2, we have seen that for the eastern spread the linear regression using Mubuga V as origin (Fig 3b) yields the speed range 0.59-1.02 km/yr (80% CL) and the regression using Kabacusi as origin yields 0.65-1.27 km/yr (80% CL). For this reason, in Fig 4a in the main paper we have applied the overall range 0.59-1.27 km/yr (80% CL). Here, instead of a 80% CL, we use a 95% CL. This yields wider ranges, as it should, namely 0.46-1.15 km/yr (95% CL) for Mubuga V and 0.46-1.46 (95% CL) for Kabacusi. Therefore, the overall range is 0.46-1.46 km/yr (95% CL). Fig I, panel a is the same as Fig 4a in the main paper, but using the 95% CL range (0.46-1.46 km/yr) instead of the 80% CL one (0.59-1.27 km/yr). Since the curves are the same as in Fig 4a, the higher upper bound for the observed speed (1.46 km/yr in Fig I, panel a, compared to 1.27 km/yr in Fig 4a) leads to a higher value for the maximum cultural transmission intensity ($C\leq2.8$ in Fig I, panel a, compared to $C\leq1.1$ in Fig 4a). Applying that $C\leq2.8$ (vertical line) in Fig I, panel b, we obtain the range 0-37% for the cultural effect. As expected, this is wider than the result from Fig 4b (0-28%). The important point is that, in both cases, the maximum cultural effect (37% from Fig I, panel b, and 28% from Fig 4b) is below 50%. Thus we reach the same conclusion, namely that the spread eastwards was mainly demic, independently of whether we use a 80% CL (Fig 4) or a 95% CL (Fig I) to estimate the observed speed range.


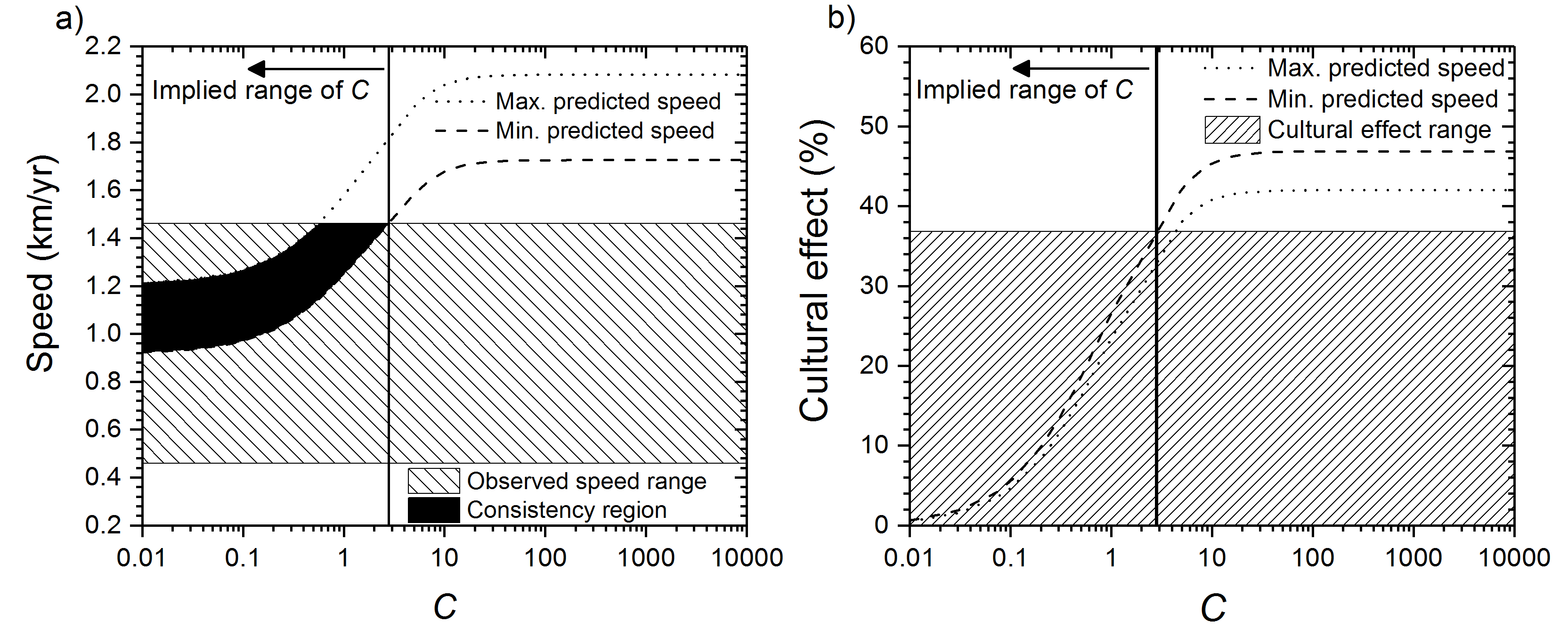


**Fig I**. Eastern spread. This Fig is the same as Fig 4 in the main paper, but using a 95% CL for the observed speed range instead of a 80% CL.

## S1 Text references

[1] Russell T, Silva F, Steele J. Modelling the Spread of Farming in the Bantu-Speaking Regions of Africa: An Archaeology-Based Phylogeography. PLoS One. 2014; 9: e87854.

[2] Philipson DW. The later prehistory of the Eastern and Southern Africa. London: Heinemann Educational Books; 1977.

[3] Huffman TN. Archaeology and ethnohistory of the African Iron Age. Ann Rev Anthropol. 1982; 11: 133-150.

[4] Denbow J. The Archeology and ethnography of Central Africa. Cambridge: Cambridge University Press; 2014.

[5] Ehret C. An African Classical Age. Eastern and Southern Africa in world history, 100B.C. to A.D.400. Oxford: James Currey; 1998.

[6] Ehret C. Bantu history: Big advance, although with a chronological contradiction. Proc Natl Acad Sci. 2015; 112: 13428-13429.

[7] Shoenbrun DL. A green place, a good place. Agrarian change, gender, and social identity in the Great Lakes region to the 15th century. Oxford: James Currey; 1998.

[8] Yamasaki F, HamadaC, Hamada TJ. Riken Natural Radiocarbon Measurements VIII. Radiocarbon. 1974; 16: 331-357.

[9] Derricourt RM. The Chronology of Zambian Prehistory. Transafrican Journal of History. 1976; 5: 1-31.

[10] Fagan, BM. Radiocarbon Dates for Sub-Saharan Africa: VI. The Journal of African History. 1969; 10: 149-169

[11] Vogel JC, Fuls A, Visser E. Pretoria Radiocarbon Dates III. Radiocarbon, 1986; 28: 1133-1172.

[12] Huffman TN. The stylistic origin of Bambata and the spread of mixed farming in southern Africa. Southern African Humanities. 2005; 17: 57-79.

[13] Stauder J. The Majangir: Ecology and Society of a Southwest Ethiopian People. Cambridge: Cambridge Univ Press; 1971.

[14] Fort J, Jana D, Humet JM. Multidelayed random walks: Theory and application to the Neolithic transition in Europe. Phys Rev E. 2004; 70: 031913.

[15] Ammerman AJ, Cavalli-Sforza LL. The Neolithic Transition and the Genetics of Populations in Europe. Princeton: Princeton University Press; 1984.

[16] Isern N, Fort J, Pérez-Losada J. Realistic dispersion kernels applied to cohabitation reaction-dispersion equations. J Stat Mech. 2008; 2008: P10012.

[17] Cavalli-Sforza LL, Bodmer WF. The Genetics of Human Populations. New York: Dover; 1999.

[18] Olson JS. The peoples of Africa: An ethnolinguistic dictionary. Wesport: Greenwood Press; 1996.

[19] Cavalli-Sforza, LL (ed.). African Pygmies. Orlando: Academic Press; 1986.

[20] Watts SJ. Marriage Migration, A Neglected Form of Long-Term Mobility: A Case Study from Ilorin, Nigeria. The International Migration Review. 1983; 17: 682-698.

[21] Bascom WR. Yoruba food. Africa. 1951; 21: 41-53.

[22] Crognier E. Marriages, migrations and the biological population in a Sara tribe from Chad. J Hum Evol. 1977; 6: 159-168.

[23] Crognier E. Biometric data on the nutritional state of a tropical African population: the Sara of Chad. Biometrie Humaine. 1969; 4: 37-55.

[24] Ehret C. Linguistic inferences about early Bantu history. In: Ehret C Posnansky M, editors. The Archaeological and Linguistic Reconstruction of African History. Berkeley: University of California Press; 1982, pp 57-65.

[25] Philipson, DW. African Archaeology. Cambridge: Cambridge University Press, Cambridge; 2005.

[26] Fort J. Synthesis between demic and cultural diffusion in the Neolithic transition in Europe. Proc Natl Acad Sci. 2012; 109: 18669-18673.

[27] Jerardino A, Fort J, Isern N, Rondelli B. Cultural diffusion was the main driving mechanism of the Neolithic transition in Southern Africa.
